# Antioxidant and Wound Healing Potential of *Vitis vinifera* Seeds Supported by Phytochemical Characterization and Docking Studies

**DOI:** 10.3390/antiox11050881

**Published:** 2022-04-29

**Authors:** Tarfah Al-Warhi, Eman Maher Zahran, Samy Selim, Mohammad M. Al-Sanea, Mohammed M. Ghoneim, Sherif A. Maher, Yaser A. Mostafa, Faisal Alsenani, Mahmoud A. Elrehany, Mohammed S. Almuhayawi, Soad K. Al Jaouni, Usama Ramadan Abdelmohsen, Abeer H. Elmaidomy

**Affiliations:** 1 Department of Chemistry, College of Science, Princess Nourah bint Abdulrahman University, Riyadh 11671, Saudi Arabia; tarfah-w@hotmail.com; 2 Department of Pharmacognosy, Faculty of Pharmacy, Minia University, Minia 61519, Egypt; emanzahran84@yahoo.com; 3 Department of Clinical Laboratory Sciences, College of Applied Medical Sciences, Jouf University, Sakaka 72341, Saudi Arabia; sabdulsalam@ju.edu.sa; 4 Pharmaceutical Chemistry Department, College of Pharmacy, Jouf University, Sakaka 72341, Saudi Arabia; 5 Department of Pharmacy Practice, College of Pharmacy, AlMaarefa University, Riyadh 13713, Saudi Arabia; mghoneim@mcst.edu.sa; 6 Pharmacognosy and Medicinal Plants Department, Faculty of Pharmacy (Boys), Al-Azhar University, Cairo 11884, Egypt; 7 Department of Biochemistry, Faculty of pharmacy, Deraya University, New Minia City 61111, Egypt; sherif.ali@deraya.edu.eg (S.A.M.); mahmoud.elrehany@deraya.edu.eg (M.A.E.); 8 Pharmaceutical Organic Chemistry Department, Faculty of Pharmacy, Assiut University, Assiut 71526, Egypt; yaabdelkarem@pharm.aun.edu.eg; 9 Department of Pharmacognosy, Faculty of Pharmacy, Umm Al-Qura University, Makkah 21955, Saudi Arabia; fssenani@uqu.edu.sa; 10 Department of Medical Microbiology and Parasitology, Faculty of Medicine, King Abdulaziz University, Jeddah 21589, Saudi Arabia; msalmuhayawi@kau.edu.sa; 11 Department of Hematology/Pediatric Oncology, Yousef Abdulatif Jameel Scientific Chair of Prophetic Medicine Application, Faculty of Medicine, King Abdulaziz University, Jeddah 21589, Saudi Arabia; saljaouni@kau.edu.sa; 12 Department of Pharmacognosy, Faculty of Pharmacy, Deraya University, New Minia 61111, Egypt; 13 Department of Pharmacognosy, Faculty of Pharmacy, Beni-Suef University, Beni-Suef 62511, Egypt; abeerabdelhakium@yahoo.com

**Keywords:** *Vitis vinifera*, benzofuran, wound healing, TNF-*α*, TGF-*β*, in silico

## Abstract

This study explored the in vivo wound healing potential of *Vitis vinifera* seed extract using an excision wound model with focus on wound healing molecular targets including TGFBR1, VEGF, TNF-*α*, and IL-1*β*. The wound healing results revealed that *V. vinifera* seed extract enhanced wound closure rates (*p* < 0.001), elevated TGF-*β* and VEGF levels, and significantly downregulated TNF-*α* and IL-1*β* levels in comparison to the Mebo^®^-treated group. The phenotypical results were supported by biochemical and histopathological findings. Phytochemical investigation yielded a total of 36 compounds including twenty-seven compounds (**1**–**27**) identified from seed oil using GC-MS analysis, along with nine isolated compounds. Among the isolated compounds, one new benzofuran dimer (**28**) along with eight known ones (**29**–**36**) were identified. The structure of new compound was elucidated utilizing 1D/2D NMR, with HRESIMS analyses. Moreover, molecular docking experiments were performed to elucidate the molecular targets (TNF-*α*, TGFBR1, and IL-1*β*) of the observed wound healing activity. Additionally, the in vitro antioxidant activity of *V. vinifera* seed extract along with two isolated compounds (ursolic acid **34**, and *β*-sitosterol-3-*O*-glucopyranoside **36**) were explored. Our study highlights the potential of *V. vinifera* seed extract in wound repair uncovering the most probable mechanisms of action using in silico analysis.

## 1. Introduction

Wounds are a significant global health issue that has serious commercial and social impacts on health institutes, patients, and caregivers [1]. Wounds are divided into physical, thermal, or chemical injuries that create an opening or crack in the skin’s integrity or modify the anatomical integrity of living tissues [2]. Wound healing is graded into the following phases: Inflammation, proliferation, extracellular matrix formation, and finally remodeling [3]. To develop new tissue, fibroblasts spread during the proliferative phase and secrete many growth factors, such as vascular endothelial growth factor (VEGF) and type I collagen [4]. Ethnomedicinal investigations have searched arduously for natural remedies for wound healing [4,5].

*Vitis vinifera* Linn. (F. Vitaceae) is a climber which is woody in nature and containing coiled climbing tendrils. It bears small, pale, tender flowers, which are modified to berry fruits that vary from green to various degrees of purple black [6,7,8]. *V. vinifera* fruits, commonly known as grape, were utilized in traditional medication since ancient times [8]. In Malatya, the fruit is helpful in forming blood [9]. While in Elazığ, it is used for treatment of anemia [10]. In Pakistan, it is commonly employed as carminatives [11]. The Tuscany area uses alcoholic drinks derived from grapes for the digestive system [12]. While Cyprus uses alcohol marinade as a liniment, poultice, and mouthwash [13].

Grape skin is a valuable source of unsaturated fatty acids, crude fibers, polyphenols proanthocyanidins (flavan-3-ol oligomers units including catechin/epicatechin), minerals, flavan-3-ols (catechins and proanthocyanidins), and resveratrol (3,5,4′-trihydroxy-trans-stilbene), which are valuable by-product for antioxidant and hygienic formation [14]. It has been reported that incorporation of its flour into wheat flour improved the nutritional properties of the bakery products [14]. Topical application of grape skin was reported to speed wound healing in mice, where it showed to increase the rate of wound shrinkage and hydroxyproline composition, and lower the epithelialization stage in the considered animals [15].

*V. vinifera’s* fruits are considered a major source of phenolic antioxidant compounds, including resveratrol, quercetin, catechins, epigallocatechin, epigallocatechin-3-gallate, procyanidins, and anthocyanins [16]. Researchers reported that applying a high-resveratrol *V. vinifera* fruit extract to the wounds of mice accelerated wound healing, which might be due to the manipulation of redox-sensitive processes that promote dermal tissue repair [17]. *V. vinifera’s* seeds consist of polyphenolic compounds, such as (+)-catechin, (−)-epicatechin, flavanols, resveratrol, and proanthocyanidins [18], that have been established to exhibit powerful anti-inflammatory, and antioxidant, anti-diabetic, anti-platelet, anti-cholesterol, anti-aging, anti-microbial, and anti-tumour properties [18]. Researchers reported that the surface application of oil of grape seed has proved to stimulate wound healing in animals, especially mice, through enhancing the time of wound shrinkage, hydroxyproline matter, and reducing the epithelialization lifetime [19].

Despite the existence of studies describing the wound healing potential of *V. vinifera* seeds, and other related organs, very little is known regarding its mode of action in wound healing potential. Consequently, our study explores the in vivo wound healing efficacy of *V. vinifera* seed extract by excision wound model, focusing on important wound healing molecular targets including tumor necrosis factor-α (TNF-*α*), transforming growth factor-beta receptor type 1 kinase (TGFBR1), interleukin -1*β* (IL-1*β*), collagen type I, and VEGF. Additionally, a phytochemical investigation of seed extract and molecular docking of isolated compounds using TNF-*α*, TGFBR1 and IL-1*β* was performed to pinpoint the chemical molecules that contribute to the wound healing activity.

## 2. Materials and Methods

### 2.1. Plant Material, Reagents, Chemicals, Spectral Analyses, Extraction, and Fractionation of V. vinifera Seeds

Plant material, chemicals, reagents, spectral analyses, extraction, and fractionation of *V. vinifera* seeds are discussed in detail in the Supplementary Materials (Materials and Methods section, pages S2).

### 2.2. In Vitro Antioxidant Activity

H_2_O_2_ scavenging and SOD scavenging activity of *V. vinifera* seed crude extract were discussed in the Supplementary Materials in detail.

### 2.3. In Vivo Wound Healing Activity

Twenty-four albino rabbits (adult male New Zealand Dutch) were used. The wound healing potency of *V. vinifera* seed crude extract was assessed utilizing the excision wound model, which is discussed in detail in the Supplementary Materials with a histological study, gene expression analysis, and western blotting.

### 2.4. Preparation of the Fatty Acid Methyl Esters with GC-MS Analysis, Isolation, and Purification of Compounds

Methylation was done using concentrated sulfuric acid to obtain FAMEs. The analysis was carried out using GC-MS. While isolation and purification of compounds were carried out using different chromatographic techniques. The details are discussed in the section of Supplementary Materials.

### 2.5. Molecular Docking Studies

The details are discussed in the Supplementary Materials for the structures of all test compound drawings and the crystal structures of TNF-*α*, TGFBR1, and 1L-*β*1.

## 3. Results

### 3.1. In Vitro Antioxidant Potential of V. vinifera Seed Extract

#### 3.1.1. Hydrogen Peroxide Scavenging Power

**Figure 1:**
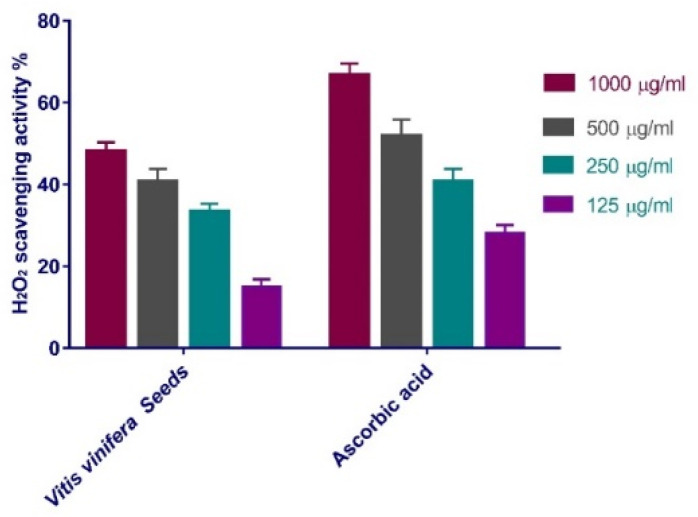
H_2_O_2_ radical scavenging activity of *V. vinifera* seed extract at variant concentrations (1000 µg/mL, 500 µg/mL, 250 µg/mL, and 125 µg/mL). Bars illustrate mean ± standard deviation (SD). Significant difference among groups is analyzed by a two-way ANOVA test.

The antioxidant power of *V. vinifera* seed extract as a scavenger potential against hydrogen peroxide (H_2_O_2_) was reported in this study. The maximal H_2_O_2_ radical scavenging activity of the seed extract of *V. vinifera* was 48.1% at 1000 µg/mL concentration, according to the data. *V. vinifera* seed extract suppressed the formation of hydrogen peroxide radicals in a dose-dependent mode, demonstrating a consistent antioxidant potential with IC_50_ of 175.8 μg/mL concentration (Figure 1), in comparison with standard (ascorbic acid, IC_50_ = 178.1 μg/mL).

#### 3.1.2. Superoxide Radical Scavenging Power

**Figure 2:**
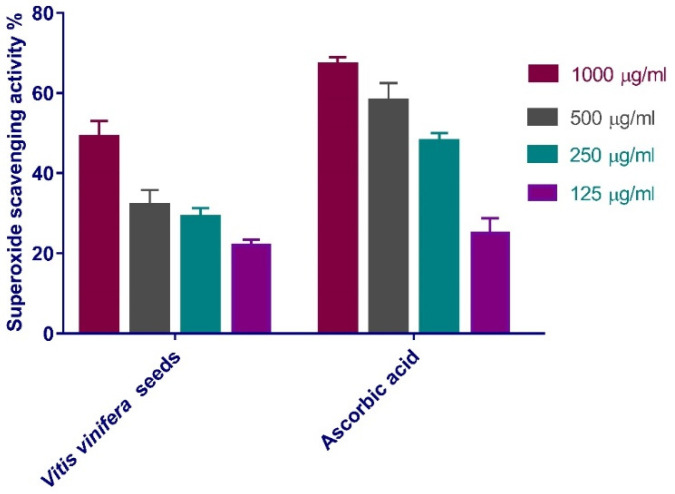
SOD scavenging power of *V. vinifera* seed extract at variant concentrations (1000 µg/mL, 500 µg/mL, 250 µg/mL, and 125 µg/mL). Bars illustrate mean ± SD (standard deviation). Significant difference among all groups is evaluated by a two-way ANOVA test.

The (SOD) potential of *V. vinifera* seed extract was evaluated. The results revealed that the scavenging impact of the standard and extract rises with concentration (Figure 2), with *V. vinifera* seed extract exhibiting the maximum SOD scavenging activity. *V. vinifera* seed extract has 49% superoxide scavenging efficacy at 1000 μg/mL concentration. IC_50_ (The concentration of *V. vinifera* seed extract required for 50% inhibition) was detected to be 151.2 µg/mL, whereas 155.8 μg/mL was needed for ascorbic acid.

### 3.2. Wound Healing Activity

#### 3.2.1. Estimation of Wound Closure Rate

**Figure 3:**
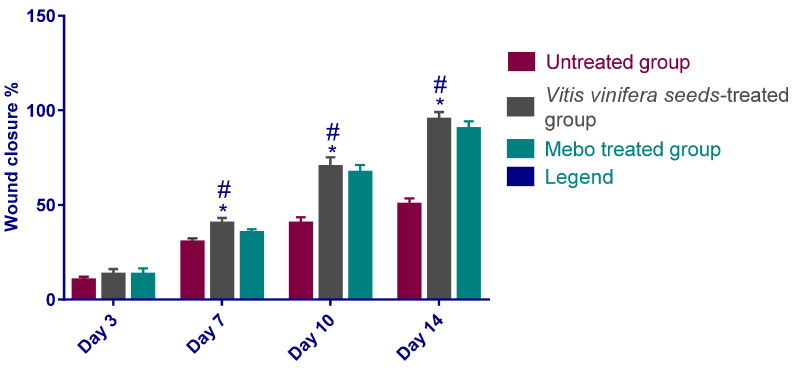
Wound closure percentages in tested groups; group 1: Untreated group (the positive control); group 2: *V. vinifera* seed-treated group; group 3: Mebo^®^-treated group, over time post-injury (0, 3, 7, 10, and 14 days). Significant difference between groups is analyzed by a two-way ANOVA test. Data were expressed as mean ± SD. * *p* < 0.001 compared with the data of the untreated group on the respective day and ^#^ *p* < 0.001 in comparison with those of the Mebo^®^ group on the respective day.

**Figure 4:**
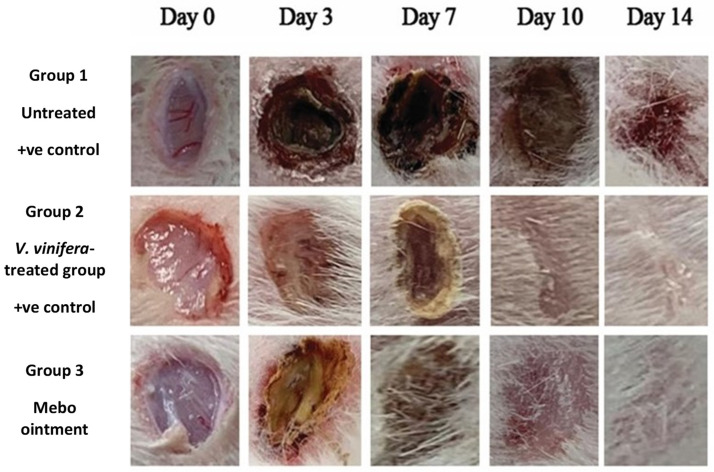
Wound closure rates in tested groups (group 1: Untreated group representing the control; group 2: *V. vinifera* seed-treated group; group 3: (MEBO^®^-treated group) over time post-injury (0, 3, 7, 10, and 14 days). Significant difference among groups is evaluated by a two-way ANOVA test. Data were expressed as mean ± SD. *p* < 0.001 compared to those of the control group on the respective day and *p* < 0.001 compared with those of the Mebo^®^ group on the relevant day.

The results show that the wound closure rate in all experimental groups amplified in a time-dependent mode. The wound closure percentages were about 10 to 13% in each group on day 3 after injury, with the smallest being in the untreated group and the highest in the treated ones, with no significant difference (*p* > 0.001) between groups. However, the wound closure in the *V. vinifera* seed-treated group reached 40% on day 7 after treatment, which was significantly higher (*p* < 0.001) than the corresponding untreated group (Figure 3 and Figure 4).

Additionally, the group that was treated with *V. vinifera* seed extract also produced high wound closure percentages compared to those of the MEBO^®^ (Moist Exposed Burn Ointment)-treated group (*p* < 0.001).

The percentage of wound closing of the *V. vinifera* seed-treated group (70%) were significantly greater (*p* < 0.001) than that of untreated group (40%) on the tenth day after injury.

On day 14, the wounds in the treated groups were perfectly cured and the wound closure scored 95% in rabbits that were treated with *V. vinifera* seed extract and 90% in the MEBO^®^-treated group (Figure 3 and Figure 4).

#### 3.2.2. Effect of Seed Extract of *V. vinifera* on Expression of TGF-*β*, TNF-*α*, IL-1*β*, Collagen Type I and VEGF

**Figure 5:**
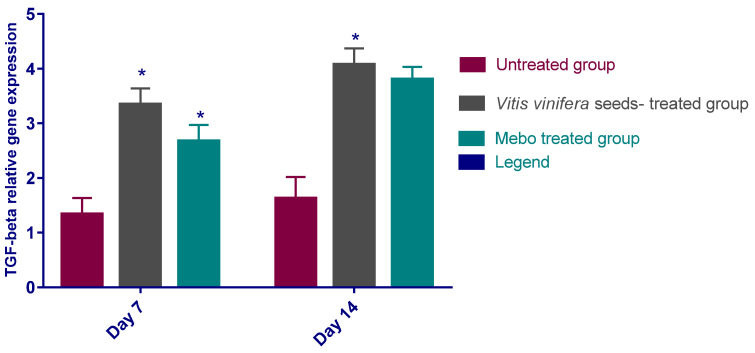
Relative gene expression of TGF-*β* in wound layers of various groups. After making normalization to glyceraldehyde 3-phosphate dehydrogenase (GAPDH), the data indicate a fold difference in expression relative to the normal control group. The bars reflect the mean ± SD and are based on the results of three independent investigations. A one-way ANOVA test is utilized to measure whether there is a significant variation between groups, where: * *p* < 0.001 when compared to the untreated group on a specified day, and *p* < 0.001 when compared to the Mebo^®^ group on the same day.

Figure 5 depicts the mRNA expression of TGF-*β* following excisional wound therapy with *V. vinifera* seed extract and MEBO^®^. TGF-*β* relative mRNA expression in skin tissues was substantially higher in *V. vinifera* seed-treated wounds for 7 or 14 days in comparison to the positive control group (*p* < 0.001).

**Figure 6:**
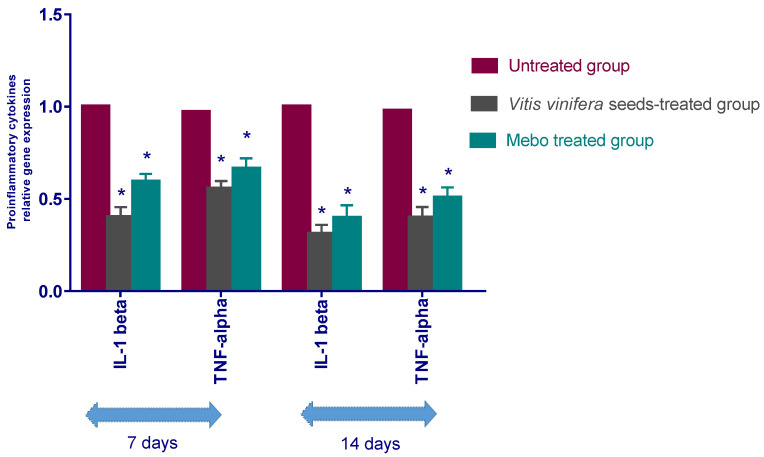
Relative gene expression of both IL-1*β* and TNF-*α* in wound tissues of various groups. After making normalization to glyceraldehyde 3-phosphate dehydrogenase (GAPDH), the data indicate a fold difference in expression relative to the natural control group. The bars indicate the mean ± SD and are based on the results of three independent investigations. A one-way ANOVA test is employed to measure whether there is a significant change between groups, where: * *p* < 0.001 when compared to the untreated group on a specified day, and *p* < 0.001 when compared to the Mebo^®^ group on the same day.

As illustrated in Figure 6, the gene expression of TNF-*α* and IL-1*β* was explained. Analysis of the gene expression of full-density wound specimens on day 7 post-injury showed that the action of the inflammatory markers’ TNF-*α* and IL-1*β* was remarkably downregulated in wounds treated with *V. vinifera* seed extract or Mebo^®^ compared to the untreated wounds. However, wounded rabbits treated with *V. vinifera* seed extract displayed a much apparent reduction in the inflammatory markers (TNF-*α*, and IL-1*β*) when in comparison to the Mebo^®^-treated group. Moreover, *V. vinifera* seed extract treatment or MEBO^®^ treatment for 14 days showed a significantly dramatic decrease in TNF-*α* and IL-1*β* mRNA expression in comparison to the untreated group at (*p* < 0.001). Again, the expressions of TNF-*α* as well as IL-1*β* in *V. vinifera* seed-treated wounds were markedly lower than in the Mebo^®^-treated group.

**Figure 7:**
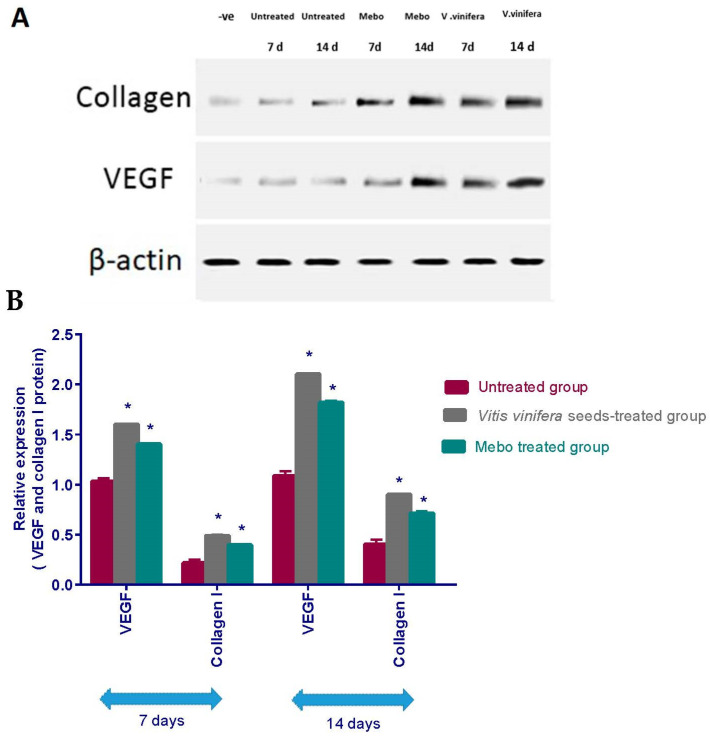
Effect of *V. vinifera* seed extract on the expression of VEGF and collagen type I proteins. (**A**) Representative immunoblotting of VEGF, collagen type I proteins, and *β*-actin proteins for all groups. (**B**) Densitometric expression of VEGF and collagen type I proteins was expressed as fold difference relative to normal control rabbits applying bands in (**A**) seeking normalization to the comparable internal control *β*-actin. The bars show the mean ± SD and are based on the results of three independent investigations. A one-way ANOVA test is applied to measure whether there is a significant difference between groups, where: * *p* < 0.001 when compared to the positive control group on a specified day, and *p* < 0.001 when compared on the same day to the Mebo^®^ group.

As illustrated in Figure 7, the relative protein expression of VEGF and type I collagen was illustrated. Analysis of the relative expression of VEGF as well as type I collagen in full thickness wound samples on day 7 post-injury showed significantly upregulated levels in wounds treated with *V. vinifera* seed extract or MEBO^®^ compared to the untreated wounds. However, wounded rabbits treated with *V. vinifera* seeds displayed a much more significant elevation in the relative protein expression compared to Mebo^®^-treated rabbits. Moreover, *V. vinifera* seed extract treatment or MEBO^®^ treatment for 14 days revealed a more significant elevation in relative protein expression when compared to untreated wounds at (*p* < 0.001). Again, the relative expression of VEGF and type I collagen genes in *V. vinifera* seed-treated wounds was markedly higher than Mebo^®^-treated wounds.

#### 3.2.3. Histopathological Investigation

**Figure 8:**
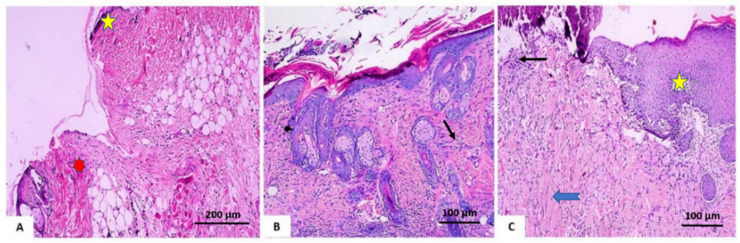
Skin wounds seven days post-incision. (**A**) Group 1 shows ordinary wounded edges with the ordinary epidermis (yellow star). The wound is packed with sloughed granulation tissue (represented by red asterisk) and blood clots. (**B**) Group 2 shows dermal collagen fibers compactly organized (black arrow). (**C**) Group 3 shows scar tissue blocking the wound (yellow star), collagen fibers (blue arrowhead) mirroring that of the neighboring normal dermis, as well as inflammatory cellular infiltration of macrophages (black arrows). (Hematoxylin and eosin stain 200 and 400).

On day 7 after treatment, group 1 (the control group) demonstrated normal wound dominance with its normal architecture, including epidermis, dermal collagen bundles, hair follicles as well as oil glands. The wound showed sloughed granulation tissue, in addition to collagen fibers compactly packed in an irregular form, inflammatory cellular infiltration, blood clots, and extravasated red blood cells. In the deepest area of the lesion, the striated muscle exposed necrotic myofiber (Figure 8A). Group 2 (*V. vinifera* seed extract-treated group), the blood clot knotted over the wound was still visible, partial reepithelization and granulation tissue occupying the injury from below was mainly cellular. Confused dense collagen with fibers developed compactly formed in an uncommon arrangement resulting in specific scarring by relation to alternative treated groups (Figure 8B). Scare tissues closing the wound and crawling of epidermal cells at wound borders were announced with limited re-epithelization in Group 3 (Mebo^®^-treated group). A significant inflammation-derived cellular infiltration (predominantly of macrophages) and collagen fibers developed, packing the defect in a reticular type with distances in between approximately nearing those of the neighbor’s natural dermis. The reticular dermis involves the typical active, enlarged, spindle-shaped fibroblasts containing the basophilic cytoplasm and oval nuclei (open face) (Figure 8C).

**Figure 9:**
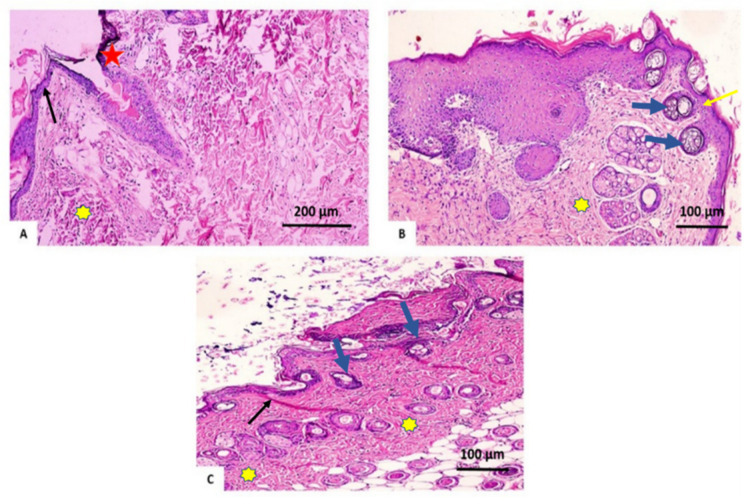
Wounded skin 14 days after incision. (**A**) Group 1 presents a large wound area with necrosis and discontinuation of the skin (red star) excessive inflammatory cells penetration included in an acidophilic matrix (yellow asterisks), and the ordinary skin (black arrow). (**B**) Group 2 shows epidermis composed of 1–3 rows of epithelial cells (yellow arrow), collagen fibers compactly established with great fibroblasts (yellow asterisk), as well as freshly developed hair follicles (blue arrows). (**C**) Group 3 illustrates normal epithelium, fine scar tissue developing into the dermis (thick black arrows), reticular dermis bears coarse collagen bundles (appearing wavy) established in various ways (yellow asterisk), as well as the freshly created hair follicles (blue arrows). (H and E stain 200 and 400).

At the post-treatment day 14, group 1 (untreated group) produced an extensive wound area and was packed with a heavy coat of granulation material, which was composed of numerous layers of connective tissue cells with inflammatory cellular infiltration included in an acidophilic matrix. The dermis is composed of confused, weak collagen with noticeable neovascularization (Figure 9A). In group 2 (*V. vinifera* seed-treated group) showed contracted scar tissue blocked the wound and the epidermis appeared formed of only 1–3 rows of epithelial cells. The granulation tissue from below was mainly cellular and populated with fibroblasts, while the reticular layer contained disorganized dense compactly arranged collagen fibers (Figure 9B). In group 3 (Mebo^®^-treated groups), the skin tissue presented more or less normal with ordinary stratified keratinized epithelium. Soft scar tissue may be found extended into the dermis. The dermal matrix offered some hair follicles, many blood capillaries, and a lack of inflammatory cells penetration. The collagen bundles in the papillary dermis are displayed as fine connecting bundles, and the reticular dermis is produced as coarse wavy bundles that appeared in diverse paths (Figure 9C).

### 3.3. Phytochemical Investigation of Vitis vinifera Crude Seed Extract

#### 3.3.1. GC/MS Analysis for Oil Content in *Vitis vinifera* Crude Extract

*V. vinifera* seeds yielded 1.20% *v*/*w* oil dry weight, marked by having no odor, being lighter than water, and yellow colored with white faint turbidity at chamber warmth. A total of 27 compounds were identified using GC/MS analysis, representing 71.16% (Table 1) of the total, and consisting of fatty acids (FA), lipids, and hydrocarbons, where fatty acids were the major item and represented about 50.33% of the oil while the hydrocarbons represented about 19.04%. Twenty-one FA were identified including fourteen saturated fatty acid (27.54%), four monounsaturated fatty acid (8.32%), and three polyunsaturated fatty acid (14.47%). The sixteen major FAs found in *V. vinifera* seeds oil were C9:0, C9:0, C14:0, C15:0, C16:1 (9), C16:0, C17:0, C18:2 (9,12), C18:2 (12,15), C18:1 (9), C18:1 (9), C18:0, C18:3 (6,9,11), C20:1 (11), C20:, and C22:1 (13) (Table 1). It was observed that palmitic, azelaic, and stearic acids were the pre-dominant SFA in *V. vinifera* seed oil, accounting for about 8.90%, 3.85%, and 3.84% of all the saturated FA, respectively. Moreover, the total UFA content was around 22.79%. Among the UFA, 9-hexadecenoic, 9-octadecenoic, and cis-11-eicosenoic acids were the pre-dominant MUFA, accounting for almost 2.57%, 2.42%, and 2.00% of the total MUFA, respectively. Combined n-2, and n-3 PUFA (18:2, C18:2, and C18:3) accounted for 14.47% of total FA, which contained 6.91, 5.72, and 1.84% of 9,12-octadecadienoic acid, 12,15-octadecadienoic acid, and 6-cis,9-cis,11-trans-octadecatrienoic acid, as major ones, respectively. Lipids represented 1.79%, which accounted mainly for 9,12,15-octadecatrienoic acid,2,3 dihydroxy propyl ester, and 9,12,15-octadecatrienoic acid,2-(acetyloxy)-1-[(acetyloxy)methyl] ethyl ester (Table 1). While hydrocarbons represented about 19.04% and included tetradecane, 1-hexadecanol, 1-docosene, and nonacos-1-ene (Table 1, Figure S1).

#### 3.3.2. Phytochemical Investigation of *V. vinifera* Seed Extract

**Figure 10:**
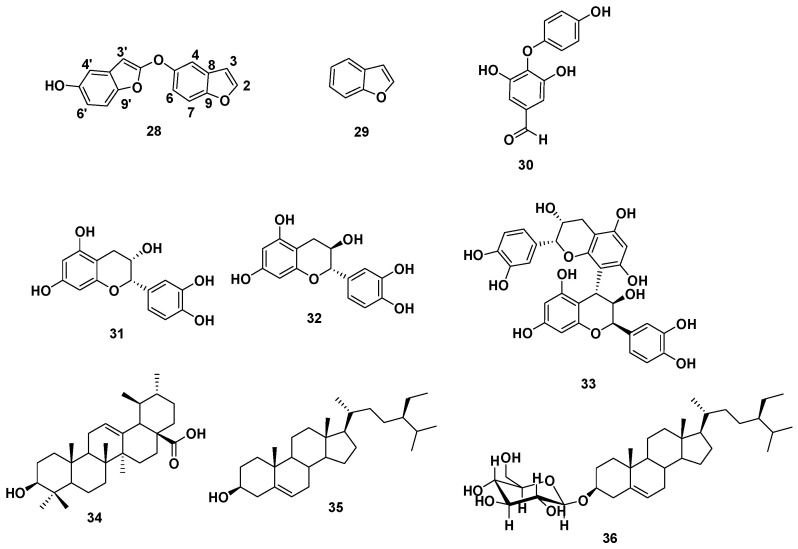
Structures of compounds isolated from *Vitis vinifera* seed extract. 2-(Benzofuran-5-yloxy) benzofuran-5-ol **28**, benzofuran **29** [20], 4-(4-hydroxyphenoxy)-3,5-dihydroxybenzaldehyde **30** [21]. (-)-*Epi*-catechin **31** [14], catechin **32** [14], procyanidin B_2_ **33** [14], ursolic acid **34** [22], *β*-sitosterol **35** [23], *β*-sitosterol-3-*O*-glucopyranoside **36** [24].

Based on physicochemical as well as chromatographic characters, the spectra obtained from UV, proton (^1^H), with DEPT-Q NMR, along with the relation to the biography and some authoritative references, the crude extract of *V. vinifera* seeds provided the new benzofuran dimer (**28**) along with eight known compounds (Figure 10).

**Figure 11:**
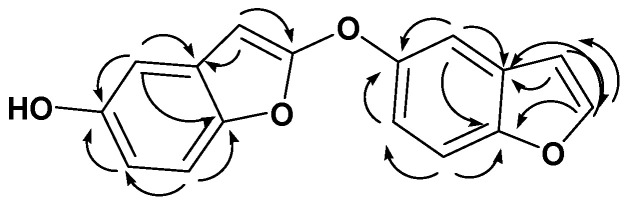
Selected **HMBC** (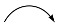)correlations of compound **28**.

Analysis of the [High Resolution Electrospray Ionization Mass Spectrometry], (HR-ESI-MS), 1D, and 2D NMR analysis data of compound **28** advocated a possible dimeric benzofuran derivative core scaffold [25]. The HR-ESI-MS data presented an adduct pseudo-molecular ion peak at *m*/*z* 267.0659 [M + H]^+^ (calc. for C_16_H_11_O_4_, 267.0657), suggesting 12 degrees of unsaturation. The ^1^H and DEPT-Q ^13^C NMR data (Table 2, Figures S2 and S3), as well as HSQC (heteronuclear single quantum correlation experiment) data (Figure S4), predicted nine methine resonance peaks appeared at *δ*_H_ 7.68, *d* (7.0) *δ*_C_ 145.9, *δ*_H_ 7.09, *dd* (3.0, 7.0) *δ*_C_ 104.0, *δ*_H_ 7.40, *d* (2.5) *δ*_C_ 108.8, *δ*_H_ 6.37, *dd* (2.5,8.0) *δ*_C_ 114.0, and *δ*_H_ 7.30, *d* (8.0) *δ*_C_ 123.8, *δ*_H_ 7.79, *d (3.0) δ*_C_ 144.5, *δ*_H_ 7.40, *d* (2.5) *δ*_C_ 108.8, *δ*_H_ 6.37, *dd* (2.5,8.0) *δ*_C_ 114.0, *δ*_H_ 7.30, *d* (8.0) *δ*_C_ 123.8, and seven quaternary carbons at *δ*_C_ 113.5, 116.8, 148.4, 148.4, 157.3, 157.3, and 160.8, indicating the characteristic core structure for dimeric benzofuran derivatives [25], where HMBC (heteronuclear multiple bond correlation) experiment of **28** (Figure 11 and Figure S5) confirmed that. The downfield shift for resonating peaks for C5, C5` at 148.4, and 160.8, suggested the presence of hydroxyl groups at C5 and C5` in each benzofuran unit.

Comparing the ^1^H and DEPT-Q ^13^C NMR data (Table 2, Figure 10), along with HSQC data (Figure S4), for C2‵ (*δ*_C_ 148.4, qC), and C3‵ ( *δ*_H_ 7.79, *d* (3.5) *δ*_C_ 144.5, CH) with C2 (7.68, *d* (7.0) *δ*_C_ 145.9, CH), and C3 (*δ*_H_ 7.09, *dd* (3.5,7.0) *δ*_C_ 104.0, CH) suggested the attachment of one of the benzofuran units at C2‵ by converting the methine of C2‵ to quaternary carbon (Table 2). The DEPT-Q ^13^C NMR data (Table 2, Figure S3) showed downfield shift for resonance peaks of C2‵ (*δ*_C_ 148.4) and C3‵ (*δ*_C_ 144.5) compared with C2 (*δ*_C_ 145.9) and C3 (*δ*_C_ 104.0), suggesting *O* attachment at C2‵. Accordingly, compound **28** was identified as 2-(benzofuran-5-yloxy) benzofuran-5-ol.

### 3.4. Molecular (In Silico) Docking Studies

In silico docking studies have been done on the crystal structures of the three main targets that might contribute to the wound healing potential of *V. vinifera* seed extract, TNF-*α*, PDB ID: 2AZ5, TGFBR1, PDB ID: 6B8Y, and IL-1*β*, PDB ID: 6Y8M, which were downloaded from the protein data bank (PDB). The binding free energy represented by (Kcal/mol) and the Root Mean Square Deviation (RMSD, Å) in the Molecular Operating Environment (MOE) program were utilized in ranking different isolated compounds in comparison to the co-crystallized ligand. Besides, the different interactions within the active sites of amino acid residues along with their energies were listed. Firstly, the docking studies within the TNF-*α* active site showed that all the test compounds attained binding energies of −3.7887 to −6.3236 kcal/mol), close to that of the co-crystallized ligand (−5.5254 kcal/mol), with an accuracy of less than 2 Å RMSD. Interestingly, the binding accuracy of these test compounds was better than the co-crystallized ligand and one of them has higher binding energy than the co-crystallized ligand (Table 3).

Virtual screening studies on TGFBR1 kinase showed interesting and promising findings. Almost all the isolated compounds (except ursolic acid **34** and *β*-sitosterol-3-*O*-glucopyranoside **36**) showed higher affinity to the active site over the co-crystallized ligand, this was presented by their better binding energy value (−4.847: −7.3066 kcal/mol) compared to the co-crystallized ligand (−5.102 kcal/mol) with an accuracy of less than 2 Å RMSD (Table 4).

All the isolated compounds showed binding free energy comparable to that of the co-crystallized ligand within the interleukin 1 beta active site with accuracy in the same way below 2 Å RMSD (Table 5). Interestingly, *β*-sitosterol-3-*O*-glucopyranoside **36** showed higher binding energy (S = −5.2574 kcal/mol) than co-crystallized ligand but its accuracy was as not high as its test congeners (>2 Å RMSD).

**Figure 12:**
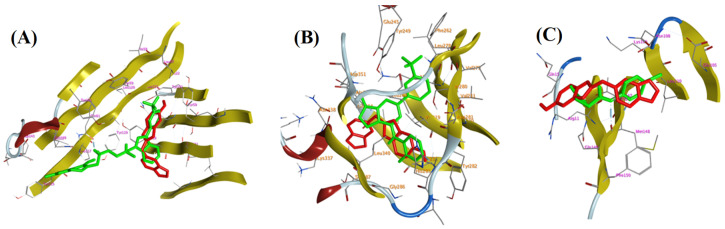
Docking poses of co-crytallized ligand (green) and compound **1** (red) within: (**A**) TNF-*α* (PDB ID: 2AZ5); (**B**) TGFBR1 kinase (PDB ID: 6B8Y); (**C**) IL-1*β* (PDB ID: 6Y8M).

Moreover, the 2D-interaction diagram Figure 12 showed a good fitting of compound **28** with various amino acid residues of three active sites comparable to the co-crystallized ligands.

**Figure 13:**
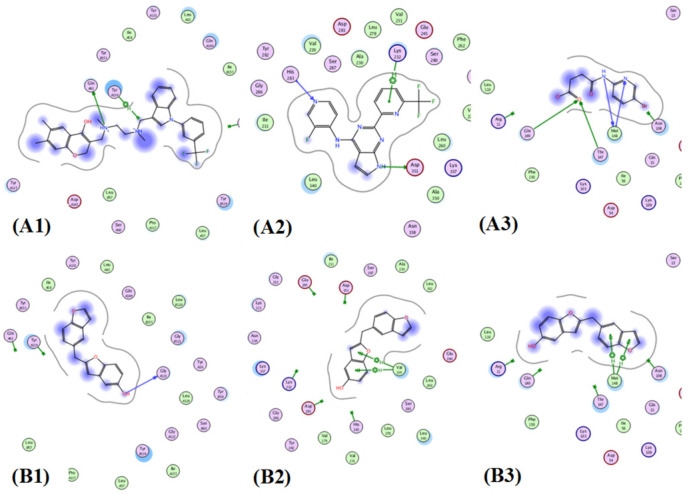
Showing 2D-binding interactions of co-crystallized ligand (**A1**–**A3**) and compound **1** (**B1**–**B3**) within active sites of: TNFα (PDB ID: 2AZ5); TGFBR1 kinase (PDB ID: 6B8Y); and IL-1*β* (PDB ID: 6Y8M).

Moreover, the 2D-interaction diagram Figure 13 showed a good fitting of compound **28** with various amino acid residues of three active sites comparable to the co-crystallized ligands; results were listed in Table 6.

### 3.5. In Vitro Antioxidant Potential of the Two Compounds Isolated from V. vinifera Seed Extract

#### 3.5.1. Hydrogen Peroxide Scavenging Activity of Ursolic Acid and *β*-Sitosterol-3-*O*-glucopyranoside

**Figure 14:**
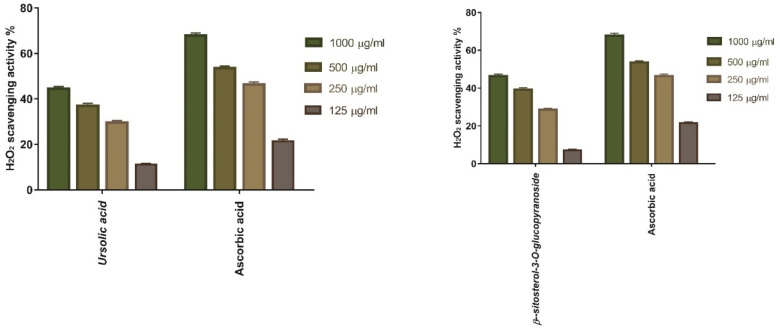
H_2_O_2_ radical scavenging activity of ursolic acid **34**, and *β*-sitosterol-3-*O*-glucopyranoside **36** at different concentrations (1000 µg/mL, 500 µg/mL, 250 µg/mL, and 125 µg/mL). Bars show mean ± standard deviation (SD). Significant difference among groups is analyzed by a two-way-ANOVA-test.

The antioxidant activities of ursolic acid **34** and *β-*sitosterol-3-*O*-glucopyranoside **36** as a scavenger potential against H_2_O_2_ were investigated in this study. The maximal H_2_O_2_ radical scavenging activities of compounds **34** and **36** were 44.44% and 46.42% at 1000 µg/mL concentration, respectively, according to the data. Compounds **34** and **36** suppressed the formation of H_2_O_2_ radicals in a dose-dependent manner, demonstrating a consistent antioxidant potential with IC_50_ of 197.1 and 222 μg/mL concentration, respectively (Figure 14), in comparison with ascorbic acid (IC_50_ = 181.2 μg/mL).

#### 3.5.2. Superoxide Radical Scavenging Activity of Ursolic Acid, and *β*-Sitosterol-3-*O*-glucopyranoside

**Figure 15:**
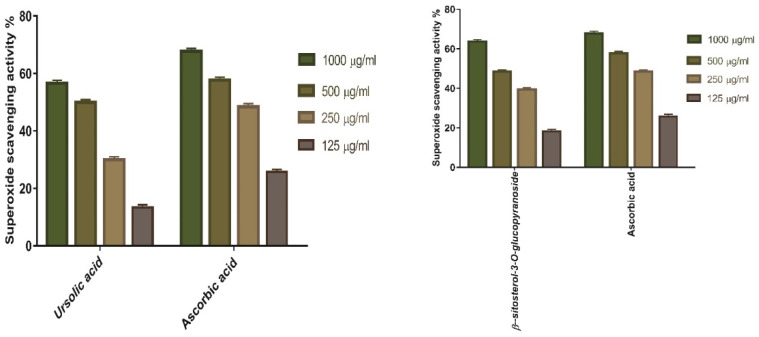
Superoxide radical scavenging potential of ursolic acid as well as *β*-sitosterol-3-*O*-glucopyranoside at variant concentrations (1000 µg/mL, 500 µg/mL, 250 µg/mL, and 125 µg/mL). Bars represent mean ± SD (standard deviation). Significant differences between groups are investigated by a two-way-ANOVA-test.

SOD activities of ursolic acid **34**, and *β*-sitosterol-3-*O*-glucopyranoside **36** was evaluated. The results showed that both compounds had 55.66%, and 63.63% SOD at 1000 μg/mL concentration, respectively. The concentration of compounds **34**, and **36** needed for 50% inhibition (IC_50_) was 221.4, and 205 µg/mL, respectively, whereas 156.6 μg/mL was demanded for ascorbic acid (Figure 15).

## 4. Discussion

Wound healing include several steps with improving tissue formation in degenerated tissue as near as possible to its real nature [26]. Studies reported that wound healing is classified into three phases: Inflammation process involving suppression of immune system and secretion of pro-inflammatory mediators, a proliferative phase through collagen growth, proliferation of fibroblasts, and development of new blood vessels, beside a remodeling phase involving regeneration and replacement of injured tissue [27,28]. Therefore, drugs that could speed wound repair with a potential contribution in all phases of the wound healing process will be good targets, specifically those with small costs and fewer side effects.

Topical application of *V. vinifera* seed extract on wounded excisions in the animals exhibited a significant (*p* < 0.001) diminishing in wound area compared to the untreated wounds (Figure 4) and that was in addition to the accelerated wound closure rate in *V. vinifera* seed extract-treated wounds. Wound closure can be characterized as the centripetal flow of the boundaries of a full-thickness wound to encourage the closure of the wound tissue [29,30,31]. Wound closure is thus a signal of re-epithelialization, angiogenesis, granulation, keratinocyte differentiation, fibroblast proliferation, and proliferation [31].

Wound-healing processes require complex interactions between cells and different growth factors [32], where the TGF-*β* affects the most important part throughout all stages of wound healing. During the inflammatory as well as hemostasis phase, the TGF-*β* recruits and stimulates inflammatory cells, macrophages, and coating neutrophils, whereas, in the proliferative-phase, it produces numerous cellular replies comprising angiogenesis, granulation tissue improvement, re-epithelialization and extracellular matrix removal [32]. It encourages fibroblasts to do proliferation and vary into myofibroblasts which cooperate in wound closure in the remodeling phase [33]. Pastar et al., 2014, and Haroon et al., 2002 [34,35], noted that chronic and non-healing wounds generally produce a failure of TGF-*β*1 warning, while Feinberg et al., 2000 [36], declared that TGF-*β*1 delivers an inhibitory effect on the interpretation of collagenases, which impair collagen and extracellular matrix. These notes are coherent with the above measurements, which established that *V. vinifera* seed extract enhanced TGF-*β*1 expression and hence recovered wound healing. When compared to untreated wound tissues, gene expression investigation of wound tissues revealed an increase in TGF-*β*1 levels in *V. vinifera*-treated wound tissues. This could indicate that *V. vinifera* seed extract increased TGF-*β*1 expression in injured tissues.

Expression of IL-1*β*, and TNF-*α* (pro-inflammatory cytokines) is required to improve neutrophils, and remove bacteria and pollution from the wound section and identifies dynamic inducer MMPs (metalloproteinase) regeneration in inflammatory and fibroblast cells. In the wound healing process, the MMP diminishes and excludes broken extracellular matrixes (ECM) to aid wound reconstruction [37]. However, a long period of the inflammatory phase draws to a complication in the healing process and these cytokines and proteinase damage the tissue and lead to the outcome of chronic wounds. TNF-*α* is one of the growth factors excreted from macrophages, which incorporates IL-1*β* to enhance and overcome respective collagen manufacture and fibroblast proliferation [38]. The TNF-*α* prompts NF-κB, which in time encourages gene interpretation of an overabundance of pro-inflammatory cytokines including TNF-*α* as well as proteases, as MMP, to clear dispersed TNF-*α* and potentiate the effects of such inflammatory cytokines [39]. So, suppressing inflammatory cytokines (TNF-*α*; and IL-1*β*) by *V. vinifera* seed extract can inhibit continued inflammation and hence avoid impaired wound repair.

Additionally, healing the wounds is resolved by various growth factors which are excreted in feedback to injury, such as VEGF, which exerts a significant role in the regenerationof new blood vessels [40]. VEGF stimulates wound healing via various processes, consisting of collagen deposition, angiogenesis, and epithelialization, and attaches to the two VEGF receptors (VEGF-1, and VEGF-2), which are revealed on vascular endothelial cells [41]. These data are coherent with the early findings that *V. vinifera* seed extract enhanced VEGF expression and hence improved wound healing. The relative protein expression of VEGF was developed in *V. vinifera* seed extract-treated wound tissues related to untreated wound tissues, which might indicate that *V. vinifera* seed extract increased VEGF expression in wound tissues.

Moreover, wound improvement is interfered in by type I collagen, which is the primary protein inside skin tissue [42] and exerts an essential role in improving connective tissue by holding tissue health and an extracellular matrix outline for cellular adhesion and migration [4]. The task of collagen in wound healing is to bring fibroblasts and facilitate the removal of modern collagen to the wound bed [43]. Chronic wounds generate enormous MMPs that obstruct the ordinary wound improvement process [43]. Collagen pickles and arrests extreme MMPs located within the extracellular matrix (ECM) [43]. So, upregulation of relative expression of Type I collagen by *V. vinifera* seed extract can thus prevent extended inflammation and hence promote wound healing.

Antioxidants are thought to help manage wound oxidative stress and hence speed up the healing process. They usually play a critical role in controlling the damage that biological components such as DNA, protein, lipids, and body tissue may sustain in the presence of reactive species. The maximal H_2_O_2_ radical scavenging activity of *V. vinifera* seed extract was 48.1% at 1000 µg/mL concentration. According to the data. *V. vinifera* seed extract suppressed the formation of H_2_O_2_ radicals in a dose-dependent manner, demonstrating a consistent antioxidant activity with IC_50_ of 175.8 μg/mL concentration (Figure 1) compared with standard ascorbic acid (IC_50_ = 178.1 μg/mL) [44]. High levels of ROS in a wound site can stimulate collagen disintegration and hence loss of ECM. When the ECM is broken, handles such as re-epithelization, and angiogenesis, which are necessary for wounds to improve, are diminished [45,46]. Moreover, elevated ROS can induce inflammation and increases pro-inflammatory cytokines and hence prolong inflammation [47].

Besides, redox signaling and enhanced oxidative stress play a vital role in normal wound healing via encouraging hemostasis, inflammation, angiogenesis, granulation tissue creation, wound closure, and extracellular matrix development and maturation [48]. As a result, the superoxide scavenging activity of *V. vinifera* seed extract was evaluated. The results showed that the scavenging impact of the standard and extract rises with concentration (Figure 2), with *V. vinifera* seed extract exhibiting the maximum superoxide radical scavenging activity. *V. vinifera* seed extract has 49% SOD efficacy at 1000 μg/mL concentration, with IC_50_ of 151.2 µg/mL, whereas 155.8 μg/mL was needed for ascorbic acid [49]. The antioxidant activity of *V. vinifera* seed extract that is attributed by its H_2_O_2_ and SOD scavenging activity can eliminate ROS and hence enhance its wound-healing activity. This antioxidant potential may be endorsed to the phenolic content of the extract.

A phytochemical investigation of the seed extract was performed to explore the chemical molecules that might contribute to the wound healing activity. Phytochemical investigation of *Vitis vinifera* extract yielded a total of 36 compounds including twenty-seven compounds (**1**–**27**) identified from seed oil using GC/MS analysis, along with nine isolated compounds, including one new benzofuran dimer (**28**), and eight known ones (**29**–**36**). The structure of the new compound was elucidated using 1D and 2D NMR and HRESIMS analyses (see Section 2.2).

Molecular docking was carried out to explore the molecular targets that might contribute to the wound healing potential. The three examined targets (TGFBR1, TNF-*α*, and IL-1*β*) play a vital role in the wound healing process. The high scores and extremely comparable interaction patterns of multiple ligands in *V. vinifera* seed extract with the stated wound healing targets provided some molecular explanation for the extract’s wound healing effect. Additionally, the binding modes and free energies obtained for the isolated compounds during the molecular docking studies within active sites of TGFBR1, TNF-*α*, and IL-1*β* (Table 3, Table 4, Table 5 and Table 6) confirm in vivo animal study results, is which manifested by the significant change in the mRNA expression of TGF-*β* (increased) and the inflammatory markers, TNF-*α* and IL-1*β* (decreased). Our data suggested that *V. vinifera* seed extract could accelerate the switching process from inflammatory to anti-inflammatory responses, which afterwards promotes healing.

## 5. Conclusions

In this study, *Vitis vinifera* seed extract displayed remarkable wound healing activity via accelerated wound closure rate, enhancing TGF-*β*1, VEGF, as well as Type I collagen expression, and suppressing inflammatory markers (TNF-*α* and IL-1*β*). Nine compounds were isolated and identified from *V. vinifera* seeds. Molecular docking analysis on three molecular targets predicted the possible mode of action in the wound healing activity. Additionally, the potent in vitro antioxidant activity of *Vitis vinifera* seed extract, and two isolated compounds (ursolic acid **34**, and *β*-sitosterol-3-*O*-glucopyranoside **36**) that are attributed by its SOD, and H_2_O_2_ scavenging activity can eliminate ROS and hence enhance its wound-healing activity. This antioxidant potential may be endorsed to the phenolic content of the extract. Finally, this study recommended the application of *V*. *vinifera* seed extract in wound care as a promising therapy to accelerate the wound healing process; however, future detailed mechanistic studies are still required to confirm those results.

## Figures and Tables

**Table 1:** *Vitis vinifera* seed oil composition using GC/MS analysis.

No.	Compound	C:D	Type	Area %	RT	RI
1	Tetradecane	C14:0	SHC	1.06	5.94	920
2	Nonanoic acid, 9-oxo-	C9:0	SFA	2.31	10.60	887
3	Octanedioic acid (Suberic acid)	C8:0	SFA	0.55	10.81	904
4	Octanoic acid, 6,6-dimethoxy-	C10:0	SFA	0.80	11.81	827
5	Undecanoic acid, 10-methyl-	C12:0	SFA	0.34	12.30	864
6	Nonanedioic acid (Azelaic acid)	C9:0	SFA	3.85	12.87	912
7	1-Hexadecanol	C16:0	SFO	2.43	13.64	943
8	Decanedioic acid (Sebacic acid)	C10:0	SFA	0.99	14.71	902
9	Tetradecanoic acid (Myristic acid)	C14:0	SFA	1.54	16.09	923
10	Undecanedioic acid	C11:0	SFA	0.34	16.54	863
11	Pentadecanoic acid	C15:0	SFA	1.30	17.87	784
12	9-Hexadecenoic acid	C16:1 (9)	MUFA	2.57	19.21	915
13	Hexadecanoic acid (Palmitic acid)	C16:0	SFA	8.90 *	19.61	939
14	1-Docosene	C22:1 (1)	MUHC	11.55 *	20.76	962
15	Heptadecanoic acid (Margaric acid)	C17:0	SFA	1.04	21.21	893
16	9,12-Octadecadienoic acid	C18:2 (9,12)	PUFA	6.91 *	22.31	923
17	12,15-Octadecadienoic acid	C18:2 (12,15)	PUFA	5.72 *	23.10	885
18	9-Octadecenoic acid	C18:1 (9)	MUFA	2.42	23.16	921
19	Octadecanoic acid (Stearic acid)	C18:0	SFA	3.84	23.33	911
20	6-Cis,9-cis,11-trans-octadecatrienoic acid	C18:3 (6,9,11)	PUFA	1.84	24.72	849
21	Cis-11-eicosenoic acid	C20:1 (11)	MUFA	2.00	25.37	848
22	Eicosanoic acid	C20:0	SFA	1.48	25.72	882
23	9,12,15-Octadecatrienoic acid,2,3 dihydroxy propyl ester	C21:3 (9,12,15)	Lipid	0.30	26.27	808
24	9,12,15-Octadecatrienoic acid,2-(acetyloxy)-1-[(acetyloxy)methyl] ethyl ester	C25:3 (9,12,15)	Lipid	1.49	26.50	817
25	Nonacos-1-ene	C29:1 (1)	MUHC	4.00	26.61	920
26	13-Docosenoic acid	C22:1 (13)	MUFA	1.33	28.04	894
27	Docosanoic acid	C22:0	SFA	0.26	28.37	852
SFA				27.54		
MUFA				8.32		
PUFA				14.47		
SHC				1.06		
MUHC				15.55		
SFO				2.43		
Lipid				1.79		
Total				71.16		

RI: retention index, RT: the retention time/minute, C:D: carbon number per double bond number-covering their position, *: main compound, SFA: saturated fatty acid, MUFA: mono unsaturated fatty acid, PUFA: poly unsaturated fatty acid, SHC: saturated hydrocarbon, MUHC: mono unsaturated hydrocarbon, SFO: saturated fatty alcohol.

**Table 2:** DEPT-Q (400 MHz) and ^1^H-NMR (100 MHz) data of compound **28** in CDCL_3_; carbon multiplicities were figured out by the DEPT-Q experiments.

Position	*δ*_C_	*δ*_H_ (*J* in Hz)
2	145.9, CH	7.68, *d* (7.0)
3	104.0, CH	7.09, *dd* (3.0, 7.0)
4	108.8, CH	7.40, *d* (2.5)
5	148.4, qC	
6	114.0, CH	6.37, *dd* (2.5,8.0)
7	123.8, CH	7.30, *d* (8.0)
8	116.8, qC	
9	157.3, qC	
2′	148.4, qC	
3′	144.5, CH	7.79, *d* (3.0)
4′	108.8, CH	7.40, *d* (2.5)
5′	160.8, qC	
6′	114.0, CH	6.37, *dd* (2.5,8.0)
7′	123.8, CH	7.30, *d* (8.0)
8′	113.5, qC	
9′	157.3, qC	

qC, quaternary, CH, methine.

**Table 3:** Interaction binding energies (S; kcal/mol) and binding accuracy (RMSD; Å) of isolated compounds from *V. vinifera* seeds and co-crystallized ligand within TNF-*α* active site (PDB ID: 2AZ5).

Compounds	Energy Score (S; kcal/mol)	RMSD (Å)
29	−3.7887	0.9620
31	−4.4254	1.2060
33	−6.3236	1.2519
34	−5.2661	1.1197
2AZ5 co−crystallized ligand	−5.5254	1.3787
28	−4.8903	1.5440
30	−4.5148	1.5722
32	−4.5435	1.8509
35	−5.3554	1.6566
36	−5.5049	1.8187

**Table 4:** Interaction binding energy (S; kcal/mol) as well as (RMSD; A) binding accuracy of isolated compounds from *V. vinifera* seeds and co-crystallized ligand within TGFBR1 kinase (PDB ID: 6B8Y).

Compounds	Energy Score (S; kcal/mol)	RMSD (Å)
28	−5.7708	0.9288
29	−4.847	0.5381
30	−6.777	0.9806
31	−5.5238	1.0294
32	−7.019	0.9637
34	6.5779	1.0290
6B8Y co-crystallized ligand	−5.102	1.1231
33	−7.3066	1.6720
35	−5.4909	1.2527
36	2.7322	2.2620

**Table 5:** Interaction binding energy (S; kcal/mol) and binding accuracy (RMSD; A) of isolated compounds from *V. vinifera* seeds and co-crystallized ligand within the IL-1*β* active site (PDB ID: 6Y8M).

Compounds	Energy Score (S; kcal/mol)	RMSD (Å)
29	−3.1641	1.0862
30	–3.6842	1.0402
32	−3.7588	1.0871
34	−4.3905	1.0397
35	−4.6213	0.8752
6Y8M co-crystallized ligand	−4.2536	1.0950
28	−3.9952	1.3281
31	−4.2309	1.1256
33	−4.2061	1.8351
36	−5.2578	2.1526

**Table 6:** Binding free energy score (S; kcal/mol) with binding interactions for different co-crystallized ligands and compound **28** within TNF-*α* (PDB ID: 2AZ5); TGFBR1 kinase (PDB ID: 6B8Y); and IL-1*β* (PDB ID: 6Y8M) binding sites.

Active Site	Ligand	Binding Energy Score (S; kcal/mol)	Ligand—Active Site Interactions		
			a. a. Residue	Bond Type	Bond Length (Å)
TNF-*α* (PDB ID: 2AZ5)	Co-crystallized ligand	−5.5254	GLN 61	H-donor	2.97
			TYR 119	H-pi	4.08
	Compound **28**	−4.8903	GLY 121	H-donor	3.11
TGFBR1 kinase (PDB ID: 6B8Y)	Co-crystallized ligand	−5.102	ASP 351	H-donor	2.72
			HIS 283	H-acceptor	2.89
			LYS 232	pi-H	3.94
	Compound **28**	−5.7708	VAL 219	pi-H	4.27
			VAL 219	pi-H	4.15
IL-1*β* (PDB ID: 6Y8M)	Co-crystallized ligand	−4.2536	MET 148	H-donor	2.73
			MET 148	H-acceptor	2.94
			THR 147	H-acceptor	2.62
			GLN 149	H-acceptor	2.46
	Compound **28**	−3.9952	MET 148	pi-H	4.51
			MET 148	pi-H	4.15

## Data Availability

Not applicable.

## Supplementary Materials

The next supporting information are available to be downloaded at: https://www.mdpi.com/article/10.3390/antiox11050881/s1, Figure S1: GC-MS spectrum for *V. vinifera* seed oil; Figures S2–S21:1D and 2D NMR spectra of compounds **28**–**36**. See [19,50,51,52,53,54,55,56,57,58].
