# Supplementary material for: Antioxidant and Wound Healing Potential of Vitis vinifera Seeds Supported by Phytochemical Characterization and Docking Studies
Source: Antioxidants (Basel). 2022 Apr 29;11(5):881. doi: 10.3390/antiox11050881 (PMC9137519; doi:10.3390/antiox11050881)
Supplement: Supplementary file 1 [file antioxidants-11-00881-s001.zip › antioxidants-1671096-supplementary.pdf]

SUPPLEMENTARY DATA

Antioxidant and Wound Healing Potential of *Vitis vinifera* Seeds Supported by Phytochemical Characterization and Docking Studies

Tarfah Al-Warhi[1,†], Eman Maher Zahran [2,†], Samy Selim [3], Mohammad M. Al-Sanea [4], Mohammed M. Ghoneim[5], Sherif A. Maher [6], Yaser A. Mostafa [7], Faisal Alsenani [8], Mahmoud A. Elrehany [6], Mohammed S. Almuhayawi[9], Soad K. Al Jaouni[10], Usama Ramadan Abdelmohsen [2,11,*] and Abeer H. Elmaidomy [12]

[1]Department of Chemistry, College of Science, Princess Nourah bint Abdulrahman University, Riyadh, Saudi Arabia

[2]Department of Pharmacognosy, Faculty of Pharmacy, Minia University, Minia 61519, Egypt

[3]Department of Clinical Laboratory Sciences, College of Applied Medical Sciences, Jouf University, Sakaka 72341, Saudi Arabia

[4]Pharmaceutical Chemistry Department, College of Pharmacy, Jouf University, Sakaka, Aljouf 72341, Saudi Arabia

[5]Department of Pharmacy Practice, College of Pharmacy, AlMaarefa University, Ad Diriyah 13713, Saudi Arabia

[6]Department of Biochemistry, Faculty of pharmacy, Deraya University, 61111 New Minia City, Egypt.

[7]Pharmaceutical Organic Chemistry Department, Faculty of Pharmacy, Assiut University, Assiut 71526, Egypt

[8]Department of Pharmacognosy, Faculty of Pharmacy, Umm Al-Qura University, Makkah 21955, Saudi Arabia

[9]Department of Medical Microbiology and Parasitology, Faculty of Medicine, King Ab-dulaziz University, Jeddah, Kingdom of Saudi Arabia

[10] Hematology/Pediatric Oncology, Yousef Abdulatif Jameel Scientific Chair of Prophetic Medicine Application, Faculty of Medicine, King Abdulaziz University, Jeddah 21589, Saudi Arabia

[11]Department of Pharmacognosy, Faculty of Pharmacy, Deraya University, New Minia 61111, Egypt

[12]Department of Pharmacognosy, Faculty of Pharmacy, Beni-Suef University, Beni-Suef 62511, Egypt

* Correspondence: usama.ramadan@mu.edu.eg

† Those authors have equally contributed to this work

Abstract

This study explored the *in vivo* wound healing potential of *Vitis vinifera*'s seeds extract using excision wound model with focus on wound healing molecular targets including TGFBR1, VEGF, TNF-α, and IL-1β. Adult male New Zealand Dutch strain albino rabbits were used. The wound healing results revealed that *V. vinifera's* seed extract enhanced wound closure rates (p<0.001), elevated TGF-β, and VEGF levels, and significantly downregulated TNF-α, and IL-1β levels in comparison to Mebo®-treated group. The phenotypical results were supported by biochemical and histopathological findings. Phytochemical investigation yielded a total of 36 compounds including twenty-seven compounds (**1-27**) identified from seed's oil using GC/MS analysis, along with nine isolated compounds. Among the isolated compounds, one new benzofuran dimer (**28**) along with eight known ones (**29-36**) were identified. The new compound structure was confirmed utilizing 1D/2D NMR, with HRESIMS analyses. Moreover, molecular docking experiments were performed to elucidate the molecular targets (TNF-α, TGFBR1 and IL-1β) of the observed wound healing activity. Additionally, the *in vitro* antioxidant activity of *V. vinifera's* seeds extract along with two isolated compounds (ursolic acid **34**, and β-sitosterol-3-*O*-glucopyranoside **36**) were explored. Our study highlights the potential of *V. vinifera's* seeds extract in wound repair uncovering the most probable mechanisms of action using *in silico* analysis.

Keywords: *Vitis vinifera;* benzofuran; wound healing; TNF-*α;* TGF-*β*; *in silico.*

1. Materials and methods

1.1. Plant material

V. vinifera seeds were collected from the Research Center for Seeds (Mallawy, Minia, Egypt) and were kindly identified by Dr. Abd ElHalim A. Mohammed (Department of Flora and Phytotaxonomy Research, Dokki, Cairo, Egypt). A voucher specimen (2021-BuPD 77) was deposited at the Department of Pharmacognosy, Faculty of Pharmacy, Beni-Suef University, Egypt.

1.2. Chemicals and reagents

The solvents utilized in this trade consisted of n-hexane (n-hex., boiling point b.p. 60–80 °C), dichloromethane (DCM), ethyl acetate (EtOAc), n-butanol (n-but.), ethanol, and methanol (MeOH) were acquired from El-Nasr Company for Pharmaceuticals and Chemicals (Egypt). High-Performance Liquid Chromatography (HPLC) and deuterated solvents employed for chromatographic and spectroscopic investigations were acquired from Sigma-Aldrich (Saint Louis, Missouri, USA), covering HPLC-methanol, HPLC-water, HPLC-acetonitrile, chloroform-d ($CDCl_3$), methanol-d_4 (CD_3OD), and dimethyl sulfoxide-d_6 (DMSO-d_6). Column chromatography (CC) was done employing silica gel 60 (63–200 µm, E. Merck, Sigma-Aldrich), and Sephadex LH-20 (0.25–0.1 mm, GE Healthcare, Sigma-Aldrich), while silica gel GF254 for Thin-layer chromatography (TLC) (El-Nasr Company for Pharmaceuticals and Chemicals, Egypt) was utilized for vacuum liquid chromatography (VLC). Thin-layer chromatography (TLC) was driven out accepting pre-coated silica gel 60 GF254 plates (E. Merck, Darmstadt, Germany; 20 × 20 cm, 0.25 mm in thickness). Spots were visualized by splashing with P-anisaldehyde (PAA) reagent (85: 5: 10: 0.5 absolute EtOH: sulfuric acid: G.A.A.: para-anisaldehyde), accompanied by heating at 110 °C [1]. For the biological course, Ketalar; (Parke-Davis, Morris Plains, NJ; 35 mg/kg I.M.) and xylazine (Rompun; Bayer Vital, Leverkusen, Germany; 5 mg/kg I.M.).

1.3. Extraction and fractionation of V. vinifera seeds

V. vinifera seeds (1.5 kg, fresh, MC_{wb}%= 33.33%) was gathered, open-dried in shade for one month to give 1kg (MC_{db}% = 50.00%), where

Moisture content of wet weight (MC_{wb}) %= $\frac{water\ weight}{wet\ sample\ weight} \times 100$

Moisture content of dry weight (MC_{db}) %= $\frac{water\ weight}{dry\ sample\ weight} \times 100$

Later the dried seeds were finely powdered using an OC-60B/60B grinding machine (60–120 mesh, Henan, Mainland China). The powdered seeds were extracted by maceration employing 70% ethanol (5 L, 3×, seven days each) at room heat, and reduced under vacuum at 45 °C employing a rotary evaporator (Buchi Rotavapor R-300, Cole-Parmer, Vernon Hills, IL, USA) to offer 175 g crude extract (17.5% dry weight basis), Where, the yield of extract expressed on dry weight basis was calculated from the following equation: Yield (g/100 g) = (W1 × 100)/W2 where W1 is the weight of the extract residue obtained after solvent removal and W2 is the weight of dried seeds.

For *in vivo* study, 10 g crude extract was employed for the wound models by dissolving *V. vinifera* seeds extract in distilled water (2 gm of crude extract in 100 mL of distilled water) and stored at 4°C in the dark.

For phytochemical examination, 165 g crude extract was suspended in 100 mL distilled water (H_2O) and generally partitioned with solvents of various polarities (*n*-Hex., DCM, EtOAc, and *n*-but.). The organic phase in each step was separately evaporated under low pressure to provide the comparable fractions *n*-Hex (24.0 g) which mainly had oil, DCM (12.0 g), EtOAc (5.0 g), and *n*-but (12.0 g), respectively, while the remaining mother liquor was then concentrated down to give the aqueous fraction.

1.4. In Vitro Antioxidant Activity

1.4.1. Hydrogen Peroxide Scavenging Activity

The reaction with a defined amount of exogenously provided H_2O_2 was used to determine the hydrogen peroxide (H_2O_2) scavenging activity that reflects the anti-oxidative capacity of *Vitis vinifera* seeds extract. Colorimetric analysis was used to estimate the residual H_2O_2 [9]. In brief, 20 µl of the sample was mixed

with 500 µl of H_2O_2 and incubated at 37 °C for 10 minutes. After that, 500 µl of enzyme/3, 5-dichloro-2-hydroxyl-benzenesulfonate solution was added and incubated at 37 °C for 5 minutes. Colorimetrically, the intensity of the colored product was measured at 510 nm. Positive control was ascorbic acid. By comparing the percentages of H_2O_2 scavenging activity, the percentage of H_2O_2 scavenging activity was determined by comparing the results of the test with those of the control using the following formula:

$$\text{scavenging activity} = \frac{\text{A control} - \text{A sample}}{\text{A control}} \times 100$$

IC_{50} of each sample was calculated after performing the assay at four different concentrations using Graph pad prism 7 software.

1.4.2. Superoxide Radical Scavenging Activity

The superoxide anion scavenging activity was measured as described by Sreenivasan et al., 2007 [10]. The superoxide anion radicals were formed in a Tris – HCL buffer (16 mM, pH8.0) containing 90 µl of NBT (0.3 mM), 90 µl of NADH (0.936 mM), 0.1 ml of OEL extract (125, 250, 500, and 1000 g/mL), and 0.8 mL Tris – HCl buffer (16 mM, PH8.0). The reaction was initiated by adding 0.1 ml PMS solution (0.12 mM) to the mixture, which was then incubated at 25˚C for 5 minutes, and At 560 nm, the absorbance was measured. Ascorbic acid was selected as a reference. The percentage inhibition was obtained by comparing the test findings to those of the control using the formula below:

$$\text{Superoxide scavenging activity} = \frac{\text{A control} - \text{A sample}}{\text{A control}} \times 100$$

IC_{50} was calculated using Graph pad prism 7 software by performing the test at four different concentrations.

1.5. In-vivo Wound Healing Activity

1.5.1. Animal Treatment, and Ethical Statement

Twenty-four adult male New Zealand Dutch strain albino rabbits. Throughout the experiment, the rabbits were placed in polypropylene cages with unlimited access to a natural pellet diet and water ad libitum. Seven days before the study's commencement, the animals were placed in well-ventilated animal houses with normal laboratory locations including temperature (25 ± 2°C), relative humidity (44–56%) as well as day and dark cycles (12:12h.).

The Ethics Committee at the Faculty of Pharmacy, Deraya University authorized this study and stated that animals should not suffer at any stage of testing and should be kept in line with the Guide for the Care and Use of Laboratory Animals (ethical permission No: 5/2021).

1.5.2. Preparation of the Test Samples for the Bioassay, Circular Excision Wound Mode, and Experimental Design

The wound healing potency of *V. vinifera* seeds crude extract was assessed utilizing the excision wound model [2] and prepared as mentioned under *Extraction and fractionation of V. vinifera seed's* part 3.3.

According to Tramontina et al., 2002 [3], rabbit wounds were made, where intra-peritoneal injection of Ketalar® (80 mg/kg), in a nutshell, was applied to anesthetize the animals. The rabbits' back hair was thoroughly shaved, and the circular incision was made with a 5 mm biopsy punch by excising the skin on the dorsal interscapular area. The ulcer was disinfected with a sterile cotton wipe moistened with normal saline and was left undressed for the period of the trial. Twenty-four adult male New Zealand Dutch strain albino rabbits were randomly divided into 3 groups of 8 rabbits each: Group 1: untreated rabbits (positive control); Group 2: rabbits treated topically with the seeds crude extract (2 mg/wound); Group 3: rabbits treated topically with Mebo® (market reference, 100 mg/wound). For 14 days and twice daily, all treatments were topically administered, and the wounds were evaluated for healing.

1.5.3. Collection of Tissue Samples, Percentage Wound Closure Rate

On days 7 and 14, full-thickness skin biopsies of entire ulcers from all groups were collected under anesthesia. Tissue samples were sectioned into three parts for gene expression analyses, western blotting, and for histological examination after storing in formalin.

The progression of the wound area was observed using a camera (Fuji, S20 Pro, Japan) every three days until the wound had entirely been healed. The wound area was evaluated using Image J 1.49v

software (National Institutes of Health, Bethesda, MD, USA), and the wound closure rate was calculated as a percentage change in the original wound area using the following formula:

$$wound\ closure\ (\%) = \frac{(Area\ of\ wound\ on\ day\ 0\ -\ Area\ of\ wound\ on\ day\ nth)}{(Area\ of\ wound\ on\ day\ 0)} \times 100$$

Where n represented the order of the examination day, i. e., 3rd, 7th, 10th, and 14th.

1.6. Western Blotting

Tissue slices were homogenized in lysis buffer (20 mM Tris, 1 mM EDTA, 100 mM NaCl, protease inhibitors mix, and 0.5 percent Triton X-100 buffer) for setting immunoblotting, and protein concentrations were calculated utilizing the Bradford technique [9]. Tissue homogenates (50 µg of each homogenate) were set on a 12 percent sodium dodecyl sulfate-polyacrylamide gel electrophoresis (SDS-PAGE) working for 1 hour at 100 voltages. Proteins were carried to polyvinylidene fluoride (PVDF) membranes after electrophoresis. They were incubated with primary antibodies for collagen I, VEGF, and -actin (1:1000; Santa Cruz Biotechnology, Santa Cruz, CA) overnight at 4°C after closing for 1 hour in a Tris-buffered saline (TBS-T) blocking solution having 5% (w/v) nonfat milk and 0.001% tween-20. As a secondary antibody, 1:5000 dilution of horseradish peroxidase-conjugated polyclonal goat anti-rabbit immunoglobulin (Cell Signaling Technology Inc., MA, USA) was operated in stopping buffer. Chemiluminescence was applied to visualize the bands, and a raised chemiluminescence kit was utilized (ECL, GE Healthcare, Chicago, IL, USA), conforming to the manufacturer's recommendations, and noticed using a luminescent image analyzer (LAS-4000, Fujifilm Co., Tokyo, Japan). Using Image J Software, bands analogous to proteins from other groups were densitometrical accessed related to the normal control group after normalization to β-actin.

1.7. Histological Study, Gene Expression Analysis

Dorsal skin samples were gathered from all creatures and settled in buffered formalin before being handled through a graded list of alcohol and xylene then inserted in paraffin blocks. To assess the density of collagen fibers, tissue strips were cut 5-6 µm thick and colored with hematoxylin/eosin and a particular Masson's trichrome. Prepared slides were studied and photographed using the Leica Application Suite (Leica Microsystems, Wetzlar, a light microscope).

Total ribonucleic acid (RNA) was separated from skin tissues applying the TRIzol reagent (Invitrogen, USA) dealing with the manufacturer's instructions [4]. The quantity of RNA extracted was assessed spectrophotometrically using a NanoDrop 1000 (Thermo Scientific, Waltham, MA, USA). Complementary Deoxyribonucleic acid (cDNA) was reverse-transcript using 1µg total RNA by high-capacity reverse transcription kit (Thermo Scientific, USA) with oligo-dT primers.

Transcript levels were established utilizing real-time Polymerase chain reaction (PCR) with the sequence-specific primers listed in Table 1. Amplification was got out in a step one real-time PCR thermal cycler (Thermo fisher, USA) dealing with the manufacturer's instructions controlling the SYBR Green PCR Master Mix (Thermo Scientific, USA). After normalization to GAPDH as a housekeeping gene, the gene expression levels were assessed applying the comparative CT method.

Table 1. Primers used for Real time PCR.

Gene name	GenBank accession		
**IL- β1**	NC_013670.1	Forward	5'-AGCTTCTCCAGAGCCACAAC-3'
		Reverse	5'-CCTGACTACCCTCACGCACC-3'
**GAPDH**	NC_013676.1	Forward	5'-GTCAAGGCTGAGAACGGGAA-3'
		Reverse	5'-ACAAGAGAGTTGGCTGGGTG-3'
**TGF-β1**	NC_013672.1	Forward	5'-GACTGTGCGTTTTGGGTTCC-3'
		Reverse	5'-CCTGGGCTCCTCCTAGAGTT-3'
**TNF-α**	NC_013680.1	Forward	5'-GAGAACCCCACGGCTAGATG-3'
		Reverse	5'-TTCTCCAACTGGAAGACGCC-3'

1.8. Spectral analyses

Proton ^{1}H and Distortionless Enhancement by Polarization Transfer-Q (DEPT-Q) ^{13}C NMR spectra were recorded at 400 and 100 MHz, respectively. Tetramethyl silane (TMS) was used as an internal standard

in chloroform-d ($CDCl_3$), methanol-d_4 (CD_3OD), and dimethyl sulfoxide-d_6 (DMSO-d_6), using the residual solvent peak (δ_H = 7.26), (δ_H = 3.34, 4.78 and δ_C = 49.9) and (δ_H = 2.50 and δ_C = 39.5) as references, respectively. Measurements were performed on a Bruker Advance III 400 MHz with BBFO Smart Probe and a Bruker 400 MHz EON Nitrogen-Free Magnet (Bruker AG, Billerica, MA, USA). Carbon multiplicities were determined using a DEPT-Q experiment. The ultraviolet radiation (UV) spectrum in methanol was obtained using a Shimadzu UV 2401PC spectrophotometer (Shimadzu Corporation – UV-2401PC/UV-2501PC, Kyoto, Japan). HRESIMS data were obtained using an Acquit Ultra Performance Liquid Chromatography system coupled to a Synaptic G2 HDMS quadrupole time-of-flight hybrid mass spectrometer (Waters, Milford, MA, USA). HPLC chromatographic separations were conducted using an Agilent 1260 Infinity preparative pump (G1361A), Agilent 1260 diode array detector VL (G1315 D), Agilent 1260 Infinity Thermostand column compartment (G1361 A), Agilent 1260 Infinity preparative autosampler (G2260A), and a YMC-Pack ODS-A A-324 column (i.d. 10 × 300 mm, YMC, Kyoto, Japan).

1.9. Preparation of fatty acids methyl esters with GC-MS Analysis

Methylation was done using concentrated sulphuric acid to obtain FAMEs [5]. Five mg of *V. vinifera* seeds fraction I (mainly contained oil) was suspended in 1 mL n-Hex., prior to derivatization. Then, 2 mL of methanolic sulphuric acids (1% v/v) were added in vials and sealed. The sample was heated in a stoppered tube at 50 °C overnight for 16 hours to speed up the reaction process, followed by adding 2 mL of water containing sodium bicarbonate (2%: w/v) to neutralize the acids. Extractions of products were done by the addition of hexane. Evaporation to remove acids was done locally in air-conditioned at room temperature for 48 hours.

The GC-MS analysis was carried out using gas chromatography-mass spectrometry instrument stands with the following specifications, Instrument: a TRACE GC Ultra Gas Chromatographs (THERMO Scientific Corp., USA), coupled with a Thermo mass spectrometer detector (ISQ Single Quadrupole Mass Spectrometer) [6]. The GC-MS system was equipped with a TR-5 MS column (30 m x 0.32 mm i.d., 0.25 μm film thickness). Analyses were carried out using helium as a carrier gas at a flow rate of 1.0 mL/min and a split ratio of 1:10 using the following temperature program: 60 ºC for 1 min; rising at 4.0 ºC/min to 240 ºC and held for 1 min. The injector and detector were held at 210 ºC, where the diluted samples (1:10 hexane, v/v) of 1μL of the mixtures were always injected. Mass spectra were obtained by electron ionization (EI) at 70 eV, using a spectral range of m/z 40-450. Identification of the oil chemical composition was de-convoluted using AMDIS software (www.amdis.net) and identified by its retention indices (relative to n-alkanes C8-C22), mass spectrum matching to (authentic standards (when available), Wiley spectral library collection, and NSIT library database).

1.10. Isolation and purification of compounds

n-Hex (20 g) was subjected to VLC fractionation using silica gel GF_{254} (column 6 × 30 cm, 100 g). Elution was performed using n-hex. : EtOAc gradient mixtures in order of increasing polarities (0, 5, 10, 15, 20, 25, 30, 35, 40, 45, 50, 60, 80 and 100%, 250 ml each). The effluents from the column were collected in fractions (250 mL each); and each collected fraction was concentrated and monitored by TLC using the system n-Hex : EtOAC 8 : 2 and PAA reagent. Similar fractions were grouped and concentrated to provide four sub-fractions (I_1–I_4). Sub-fraction I_2 (50 mg) was further fractionated on silica gel 60 (100 × 1 cm, 20 g) and eluted using n-hex. : EtOAc isocratic mixture (1%, 500 mL) to afford compound **28** (20 mg). Sub-fractions I_3 was further fractionated on silica gel 60 (100 × 1 cm, 20 g) and eluted using n-Hex : EtOAC isocratic mixture (1%, 500 mL) to afford compound **35** (20 mg). Finally, crystallization of Sub-fractions I_4 using methanol afforded compound **34** (50 mg).

Sub-fractions II and III (1.5, 5.0 g) were separately subjected to VLC fractionation on a silica gel (column 6 × 30 cm, 50 g) and eluted using DCM : MeOH gradient mixtures in the order of increasing polarities (0, 5, 10, 15, 20, 25, 30, 35, 40, 45, 50, 60, 80 and 100%, 1 L each). The effluents were collected in fractions (100 mL each); each fraction was concentrated and monitored by TLC using the system DCM : MeOH (9.5 : 0.5) and PAA reagent. Similar fractions were grouped and concentrated to provide two sub-fractions (II_1–II_2), and one sub-fraction (III_1), which were further purified on a Sephadex LH_{20} column

(0.25–0.1 mm, 100 × 0.5 cm, 100 gm) eluted with MeOH to afford compound **29** (16 mg), **30** (12 mg) and **36** (6 mg), separately.

Sub-fraction IV (8.0 g) was subjected to VLC fractionation on a silica gel column (6 × 30 cm, 50 g) and eluted using DCM : MeOH gradient mixtures in the order of increasing polarities (0, 5, 10, 15, 20, 25, 30, 35, 40, 45, 50, 60, 80 and 100%, 1 L each). The effluents were collected in fractions, concentrated, and monitored by TLC using the system DCM : MeOH 8 : 2 and PAA reagent. Similar fractions were grouped and concentrated to provide two sub-fractions: IV_1 and V_2, sub-fraction IV_2 was further purified on C-18 RP-HPLC using H_2O-CH_3CN (10–60%, 30 min, 5 mL/min) to afford compounds **31** (20 mg), **32** (10 mg), and **33** (10 mg).

2-(benzofuran-5-yloxy) benzofuran-5-ol (1): white powder; [UV (MeOH) λ_{max} ($\log_{\varepsilon}$) 225 (5.5), 260 (6.0), 300 (4.5) nm; IR υ_{max} (KBr) 3429, 3100, 3030, 1680, 1600, 1450, 1300, 835, 601 cm^{-1}; NMR data; see Table 2; HR-ESI-MS m/z 267.0659 $[M + H]^+$ (calc. for $C_{16}H_{11}O_4$, 267.0657).

1.11. Molecular Docking Studies

The structures of all test compounds were drawn using ChemDraw® Ultra (Ver. 8), converted to smiles, transferred to Molecular Operating Environment (MOE 2014) software, and finally their energies were minimized using MMFF94x force field with a gradient RMS of 0.1 kcal/mol/A∧2. Crystal structures of TNF-α with a small molecule inhibitor (TNF-α PDB ID: 2AZ5, resolution 2.10 Å) [7], TGFBR1 domain (T204D) in complex with N-(3-fluoropyridin-4-yl)-2-[6-(trifluoromethyl)pyridin-2-yl]-7H-pyrrolo[2,3-d]pyrimidin-4-amine (TGFBR1 PDB ID: 6B8Y, resolution 1.65 Å) [8], and fragment bikini bound to 1L-β1 (1L-β1 PDB ID: 6Y8M, resolution 1.9 Å), were downloaded from RCSB Protein Data Bank (https://www.rcsb.org/). All the three proteins were prepared with MOE program LigX protocol; also, hydrogens were added to protein structure at a cutoff of 15 A. To validate the docking studies at the 3 active sites, the co-crystallized ligands were re-docked into the binding sites of each target protein. The co-crystallized ligand and compounds (**28-37**) were then docked into each active site using the triangular placement method and London δG for rescoring 1 for only 10 retained poses for each test compound, and the force field protocol for refinement rescore 2 for the same number of poses. Finally, the resultant docking poses of each test compound were visually inspected, where the ones with the lowest binding interaction energy (S; kcal/mol), and high accuracy (root mean square root RMSD; Å) were further used to investigate the 2D-binding interactions with various amino acid residue of each binding site.

1.12. Statistical Analysis

The data were presented as mean ± standard deviation (SD) (n = 8). The one-way analysis of variance (ANOVA) was used, followed by Dunnett's test. For statistical computations, Graph Pad Prism 7 was utilized (Graph pad Software, San Diego, California, USA). When p<0.001 was used, the results were considered significant.

List of Contents

Figure S1. GC-MS Chromatogram of *Vitis vinifera* seeds oil

Figure S2. ^{1}H NMR spectrum of compound **28** measured in $CDCl_3$ at 400 MHz

Figure S3. DEPT-Q NMR spectrum of compound **28** measured in $CDCl_3$ at 100 MHz

Figure S4. HSQC spectrum of compound **28** measured in $CDCl_3$

Figure S5. HMBC spectrum of compound **28** measured in $CDCl_3$

Figure S6. ^{1}H NMR spectrum of compound **29** measured in CD_3OD at 400 MHz

Figure S7. DEPT-Q NMR spectrum of compound **29** measured in CD_3OD at 100 MHz.

Figure S8. ^{1}H NMR spectrum of compound **30** measured in CD_3OD at 400 MHz

Figure S9. DEPT-Q NMR spectrum of compound **30** measured in CD_3OD at 100 MHz.

Figure S10. ^{1}H NMR spectrum of compound **31** measured in CD_3OD at 400 MHz

Figure S11. DEPT-Q NMR spectrum of compound **31** measured in CD_3OD at 100 MHz

Figure S12. ^{1}H NMR spectrum of compound **32** measured in CD_3OD at 400 MHz

Figure S13. DEPT-Q NMR spectrum of compound **32** measured in CD_3OD at 100 MHz

Figure S14. ^{1}H NMR spectrum of compound **33** measured in CD_3OD at 400 MHz

Figure S15. DEPT-Q NMR spectrum of compound **33** measured in CD_3OD at 100 MHz

Figure S16. ^{1}H NMR spectrum of compound **34** measured in DMSO-d_6 400 MHz

Figure S17. DEPT-Q NMR spectrum of compound **34** measured in DMSO-d_6 at 100 MHz

Figure S18. ^{1}H NMR spectrum of compound **35** measured in $CDCl_3$at 400 MHz

Figure S19. DEPT-Q NMR spectrum of compound **35** measured in $CDCl_3$ at 100 MHz

Figure S20. ^{1}H NMR spectrum of compound **36** measured in DMSO-d_6 at 400 MHz

Figure S21. DEPT-Q NMR spectrum of compound **36** measured in DMSO-d_6 at 100 MHz

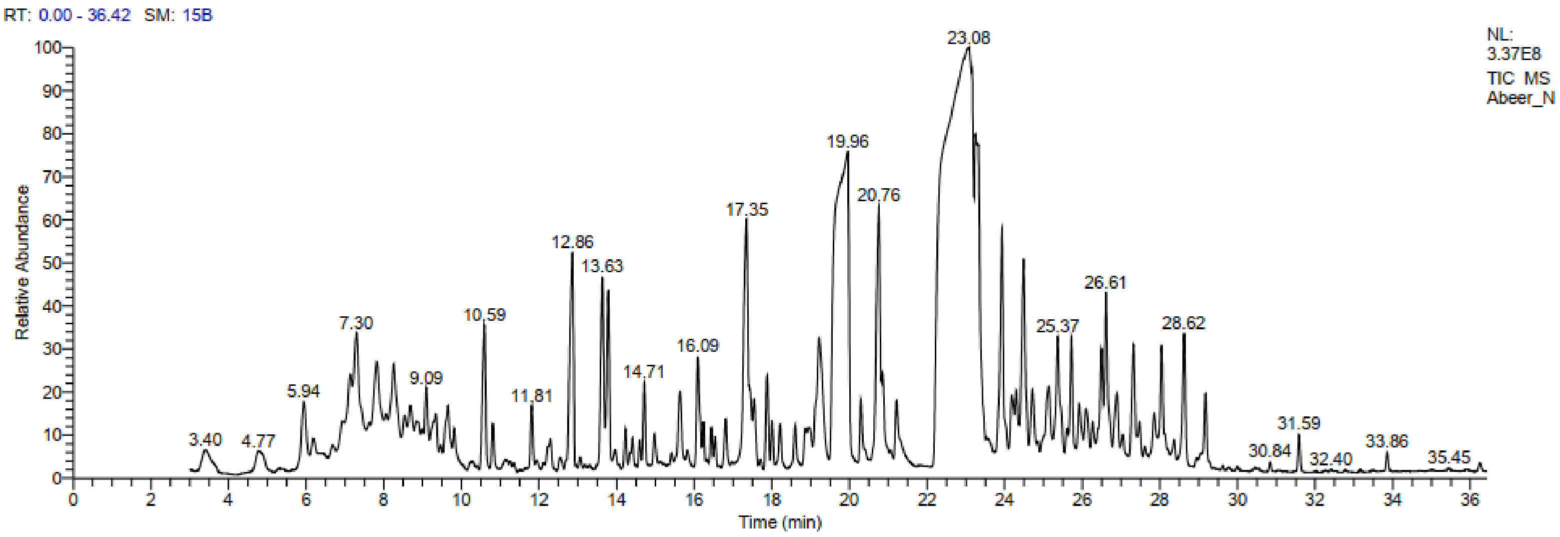

Figure S1. GC-MS Chromatogram of *Vitis vinifera* seeds oil

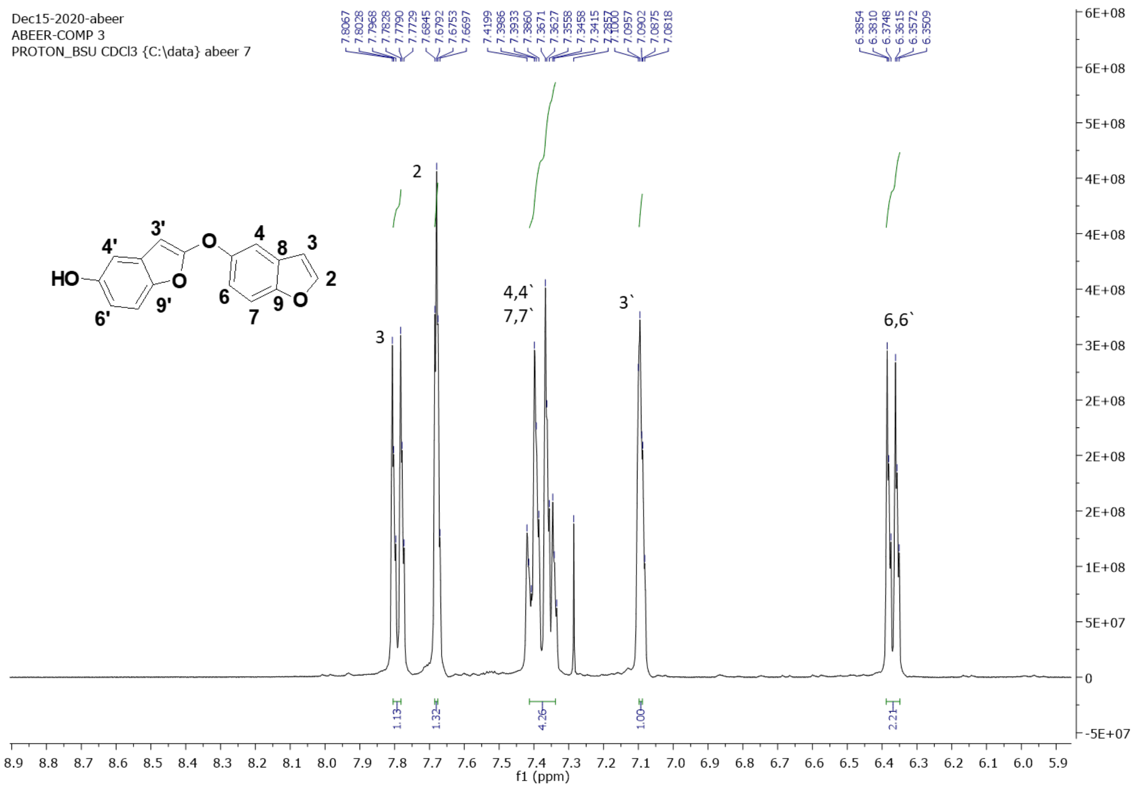

Figure S2. ^{1}H NMR spectrum of compound **28** measured in $CDCl_3$ at 400 MHz

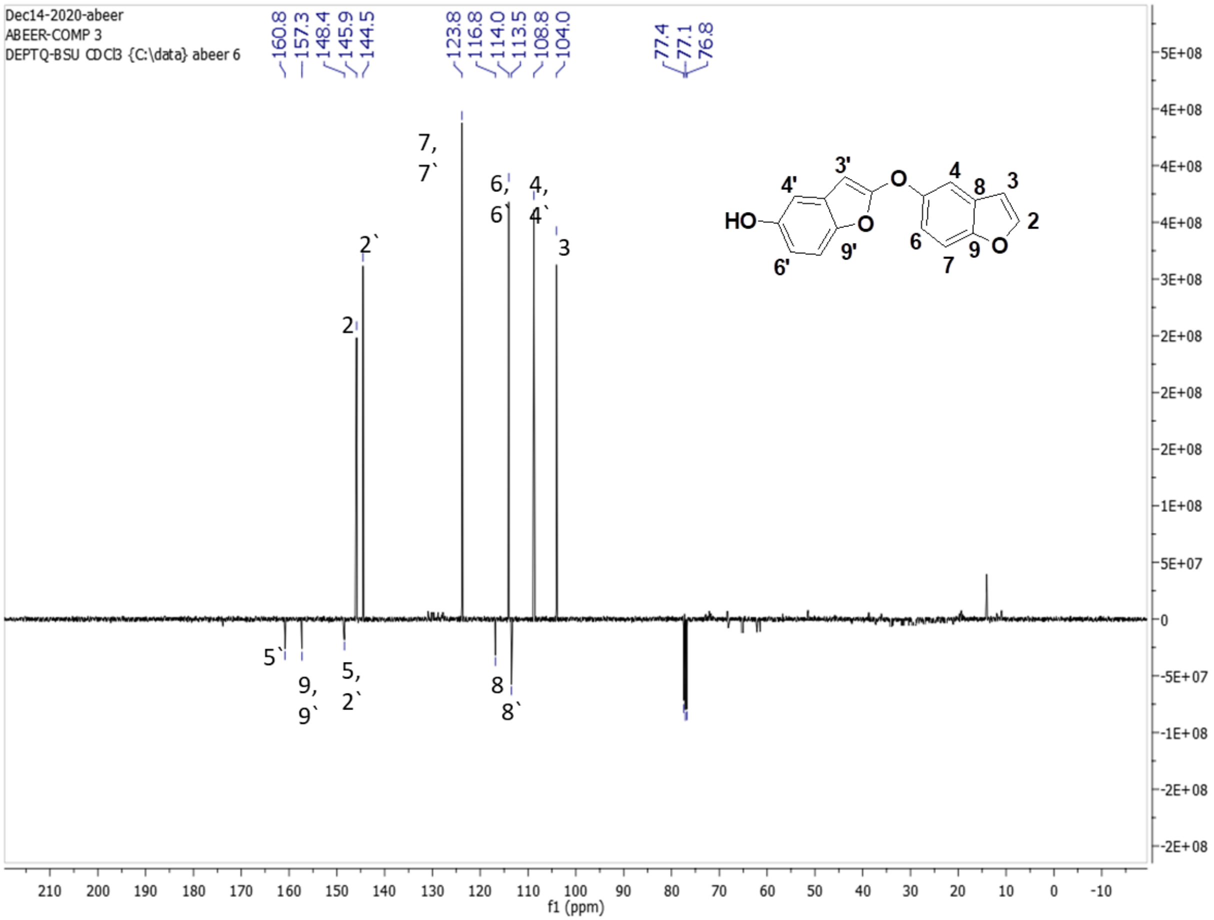

Figure S3. DEPT-Q NMR spectrum of compound **28** measured in $CDCl_3$ at 100 MHz

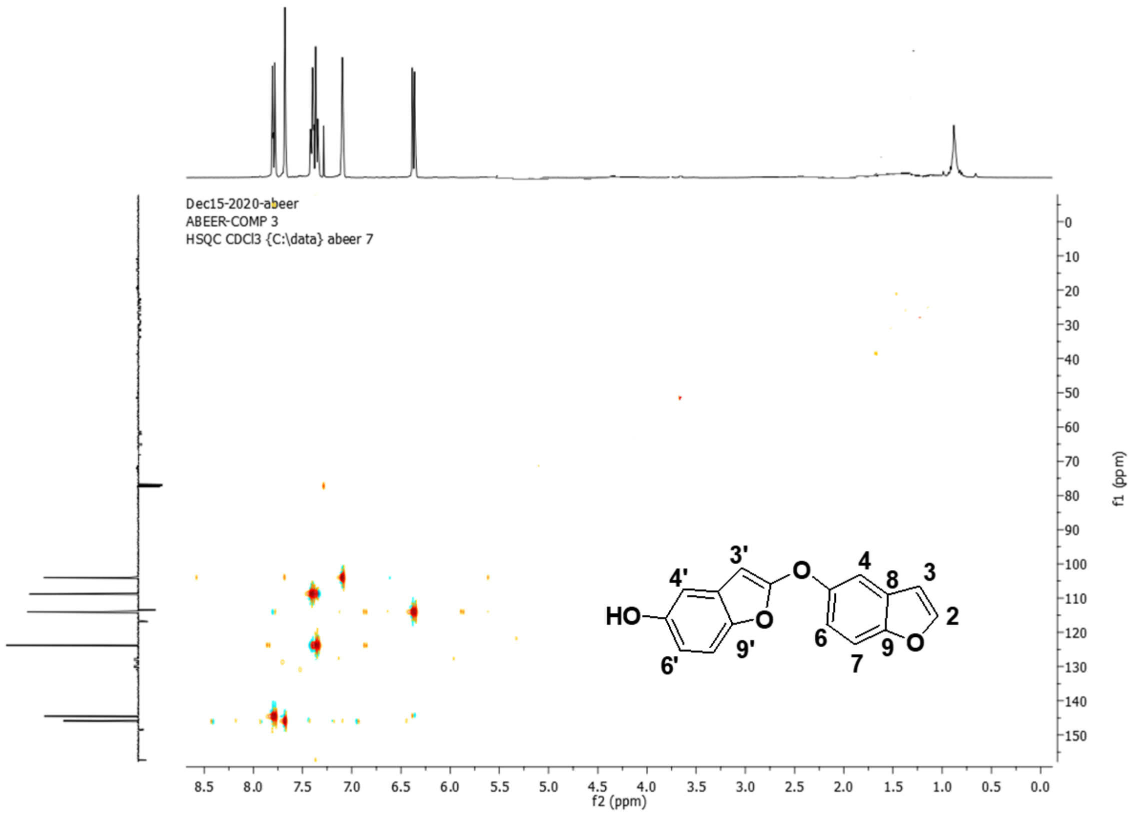

Figure S4. HSQC spectrum of compound **28** measured in $CDCl_3$

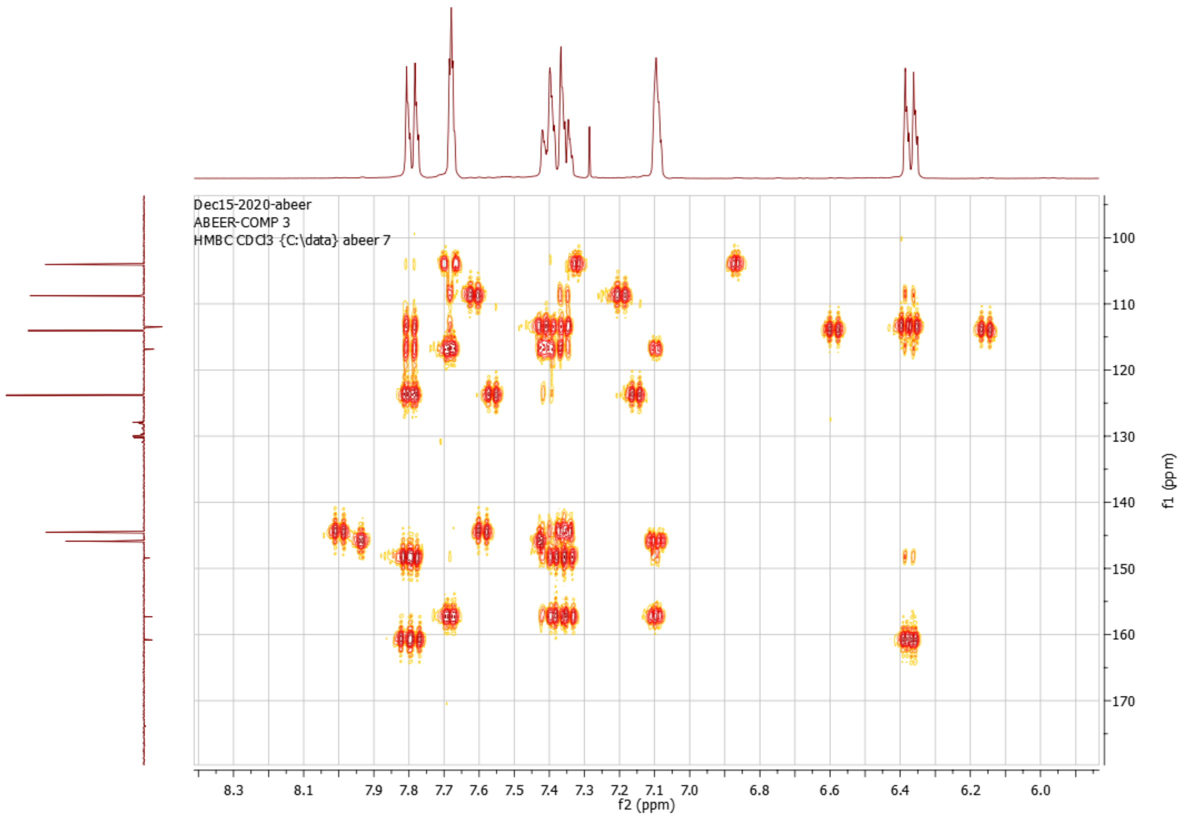

Figure S5. HMBC spectrum of compound **28** measured in CDCL$_3$-*d*

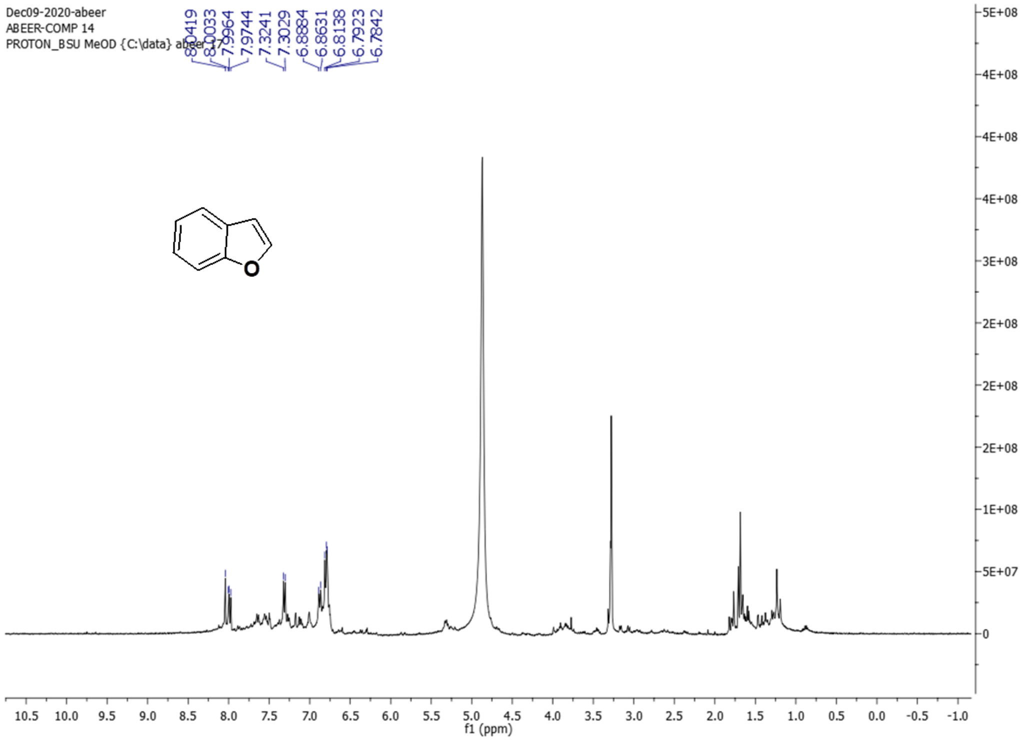

Figure S6. ^{1}H NMR spectrum of compound **29** measured in CD_3OD-d_4 at 400 MHz

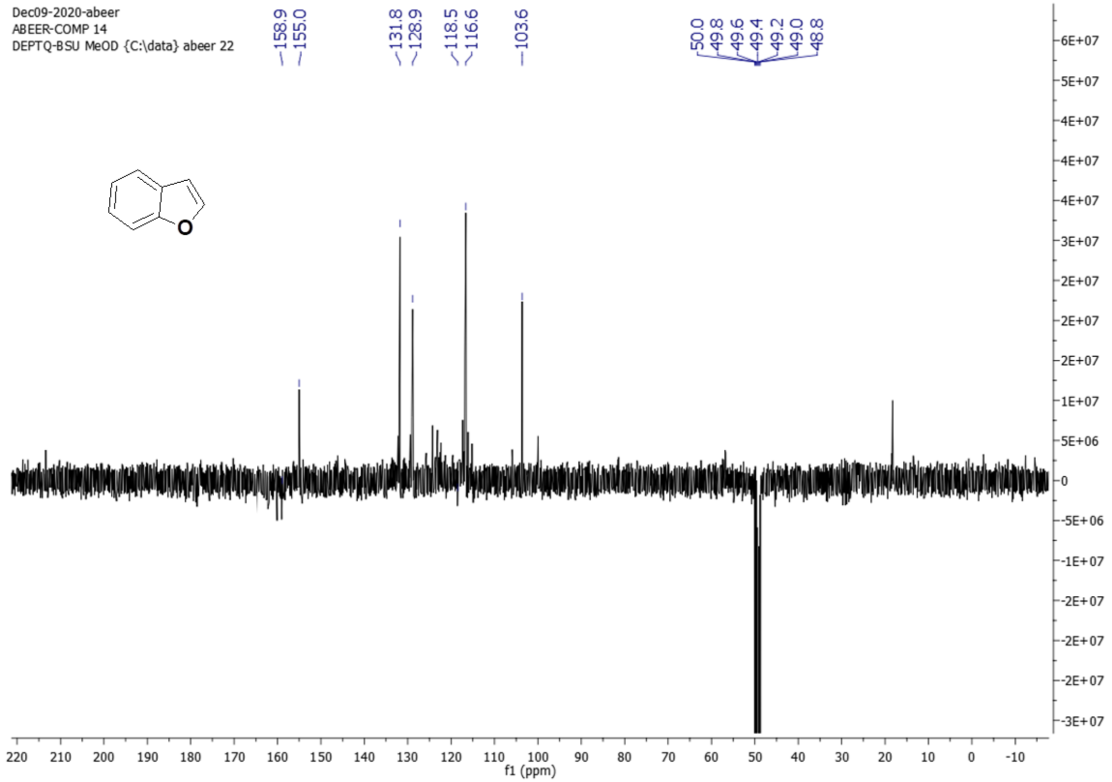

Figure S7. DEPT-Q NMR spectrum of compound **29** measured in CD_3OD-d_4 at 100 MHz.

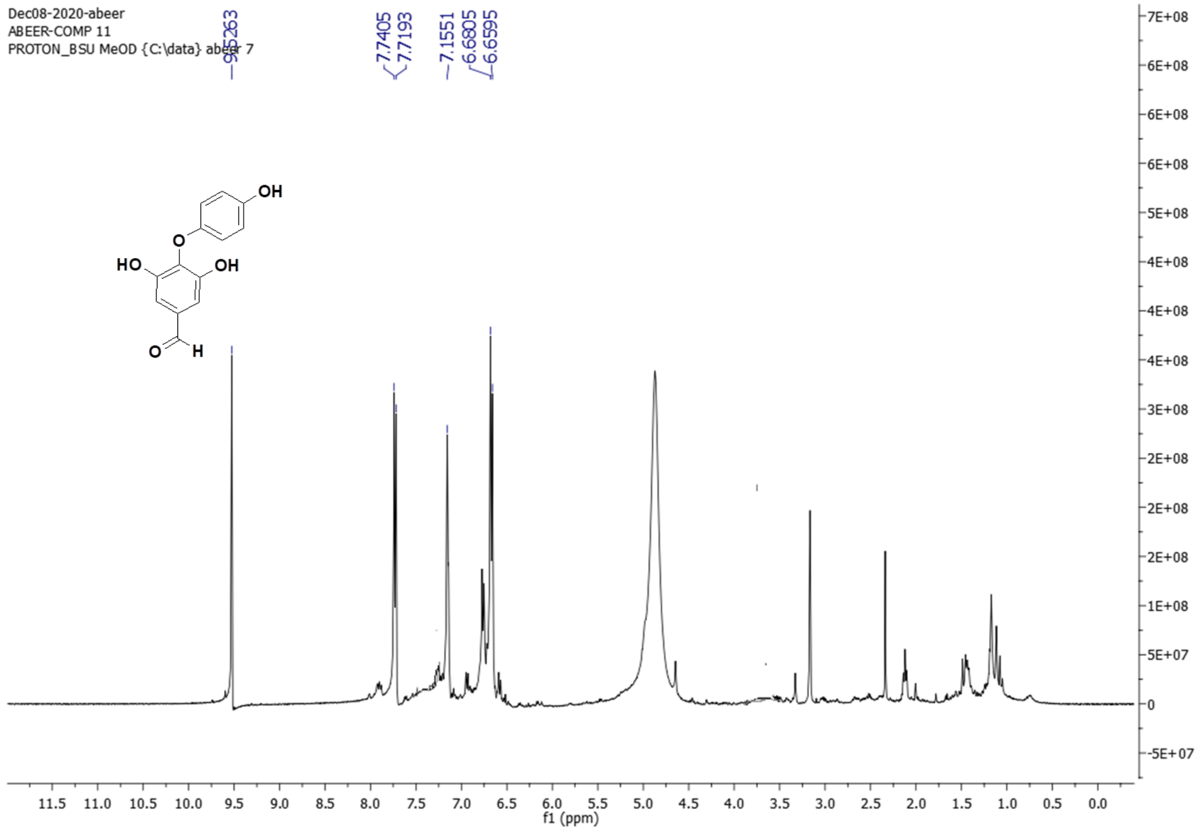

Figure S8. ^{1}H NMR spectrum of compound **30** measured in CD_3OD-d_4 at 400 MHz

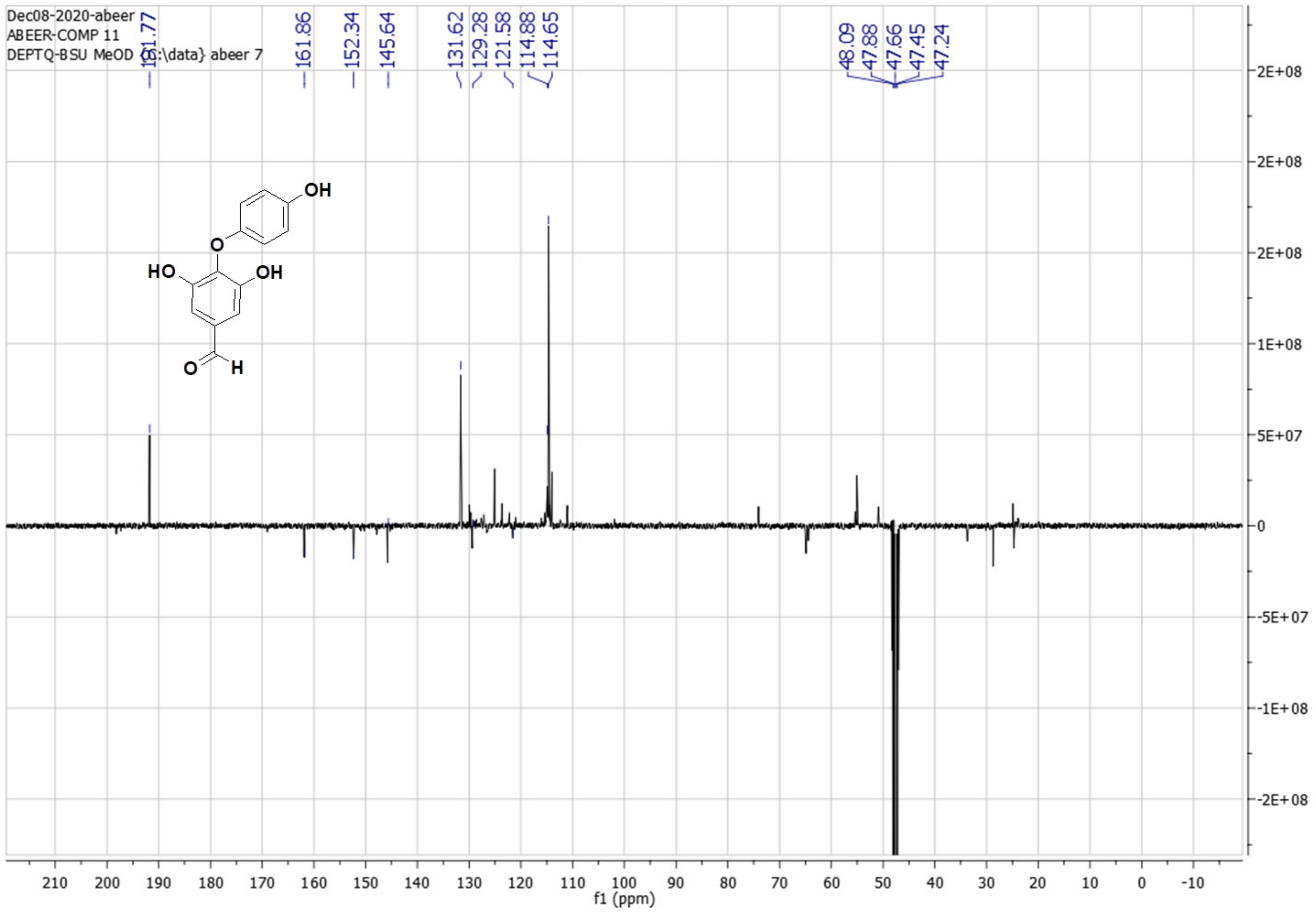

Figure S9. DEPT-Q NMR spectrum of compound **30** measured in CD_3OD-d_4 at 100 MHz.

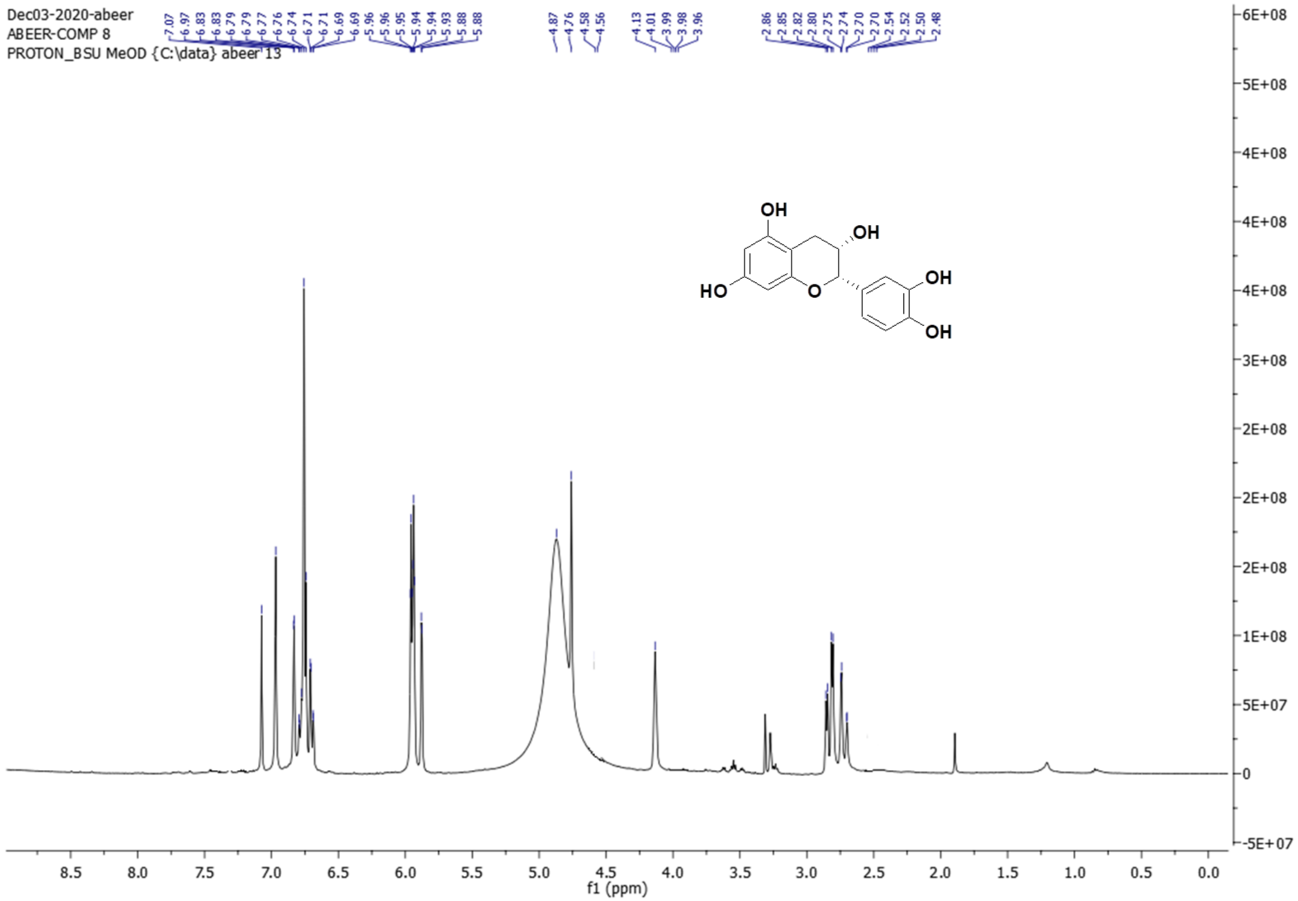

Figure S10. ^{1}H NMR spectrum of compound **31** measured in CD_3OD-d_4 at 400 MHz

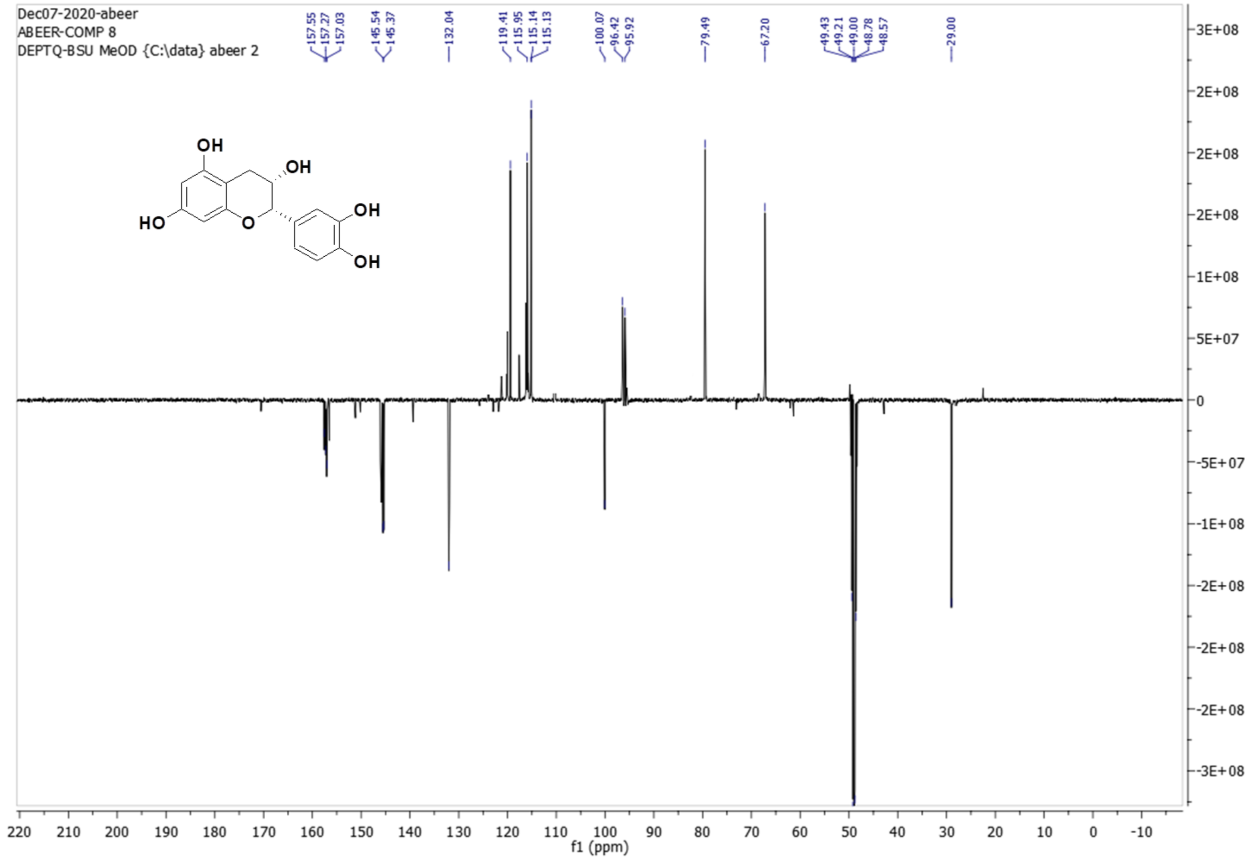

Figure S11. DEPT-Q NMR spectrum of compound **31** measured in CD_3OD-d_4 at 100 MHz

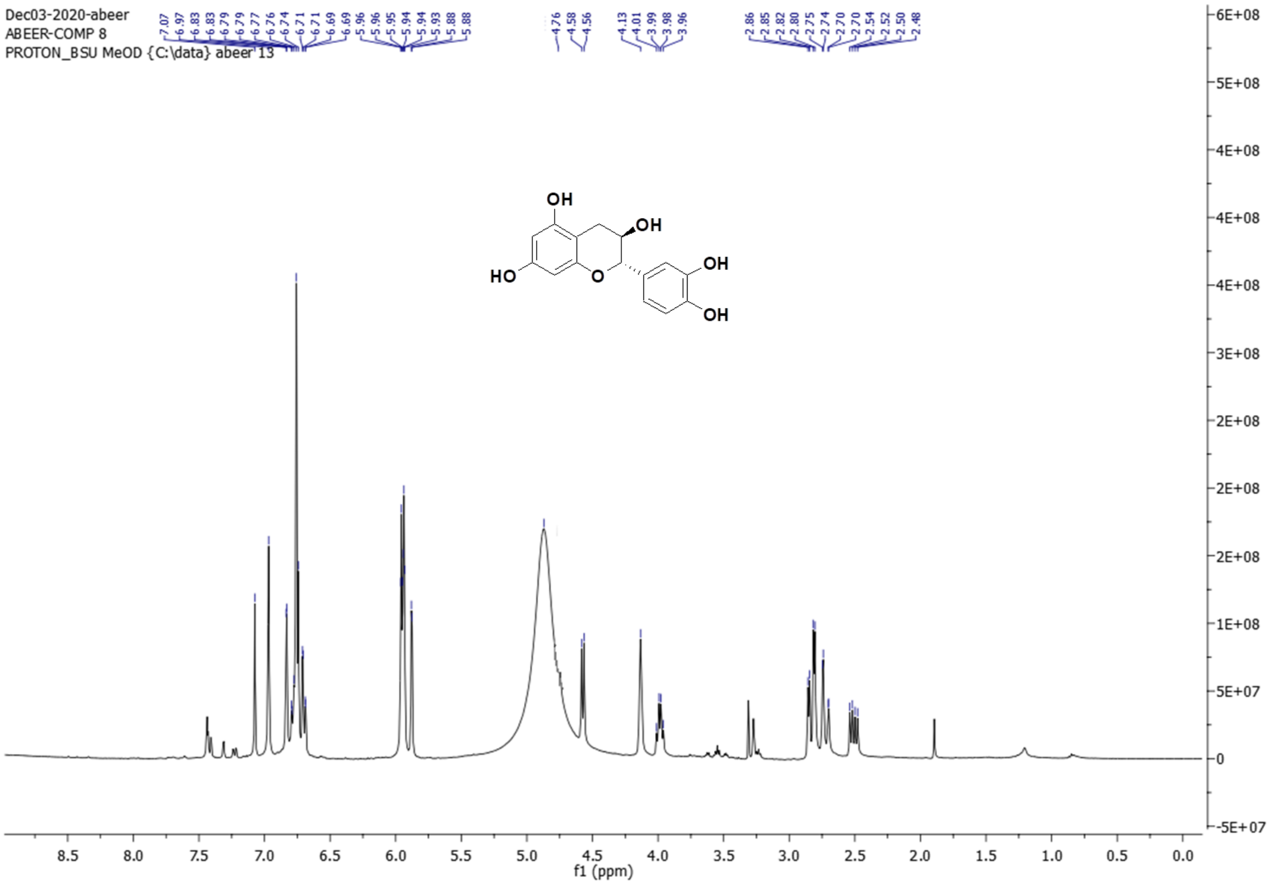

Figure S12. ^{1}H NMR spectrum of compound **32** measured in CD_3OD-d_4 at 400 MHz

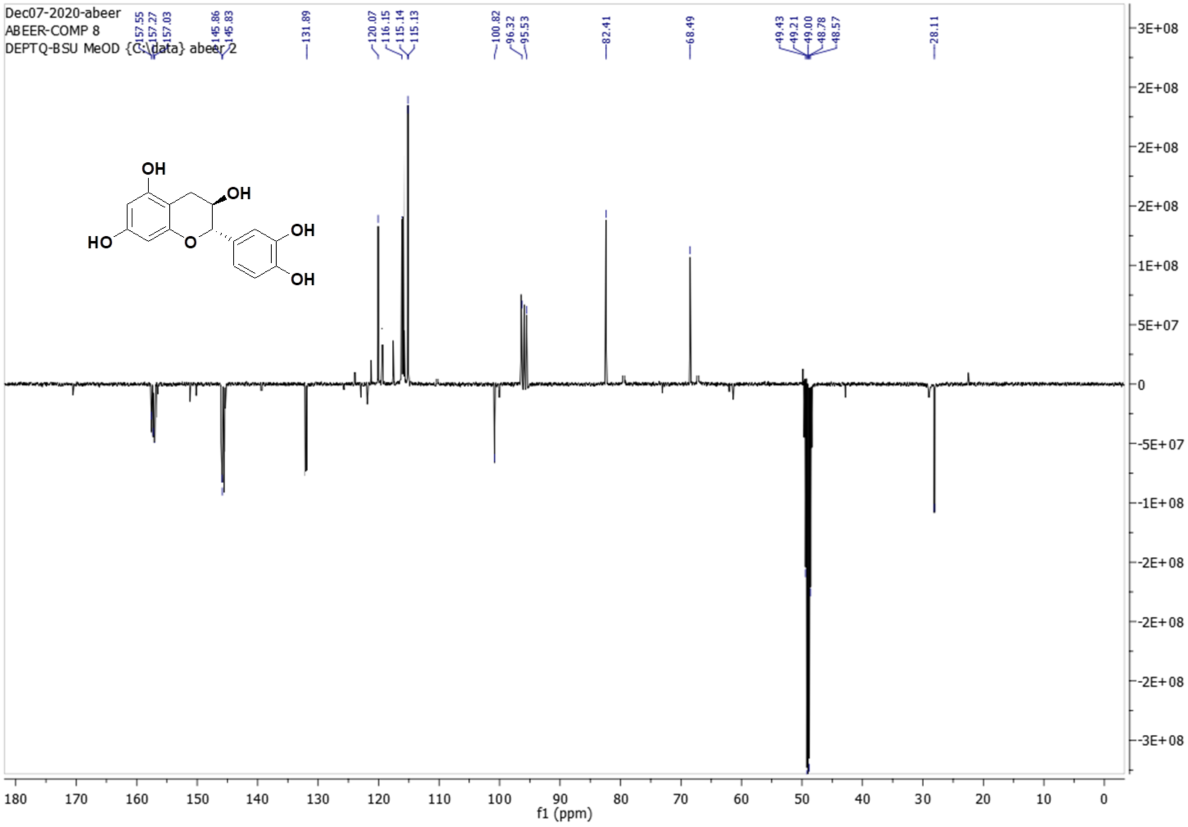

Figure S13. DEPT-Q NMR spectrum of compound **32** measured in CD_3OD-d_4 at 100 MHz

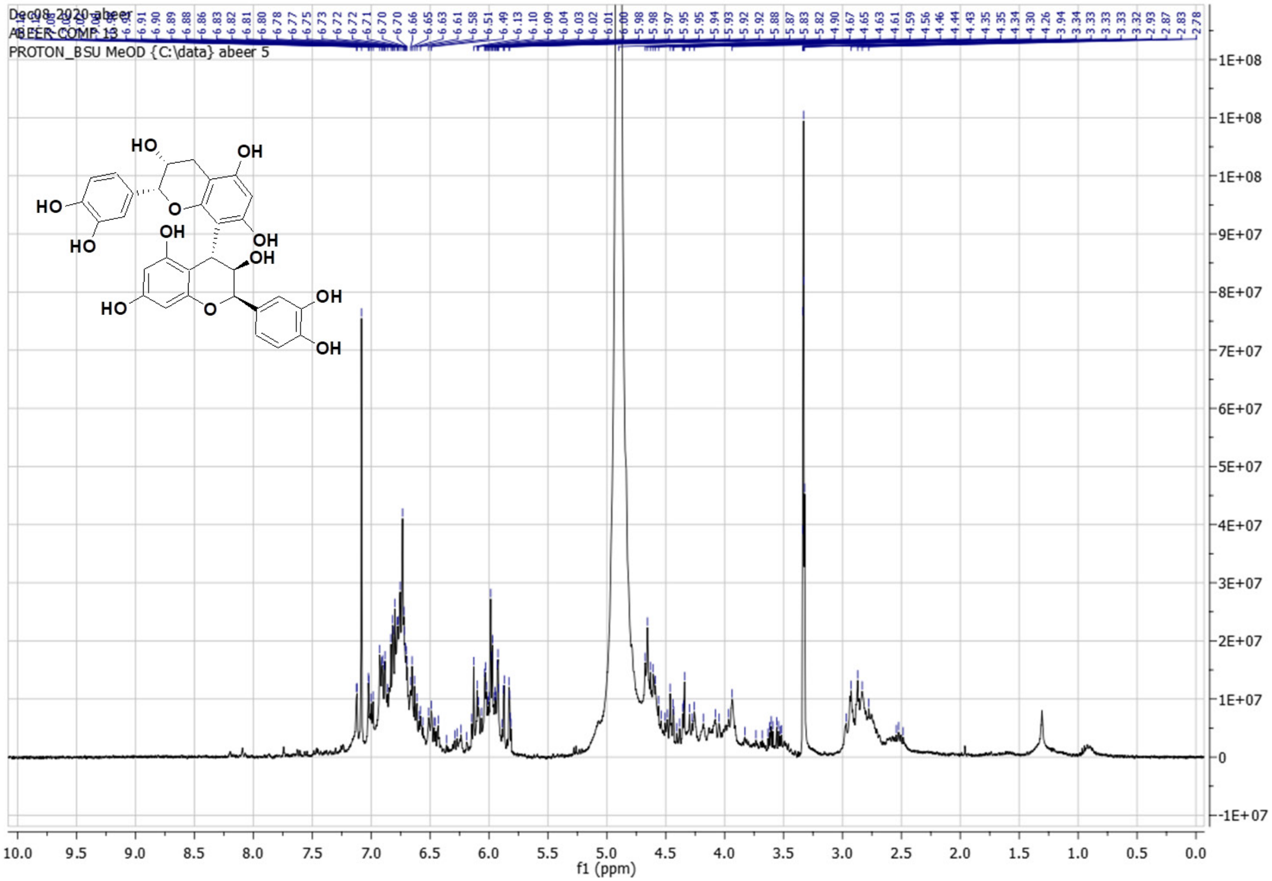

Figure S14. ^{1}H NMR spectrum of compound **33** measured in CD_3OD-d_4 at 400 MHz

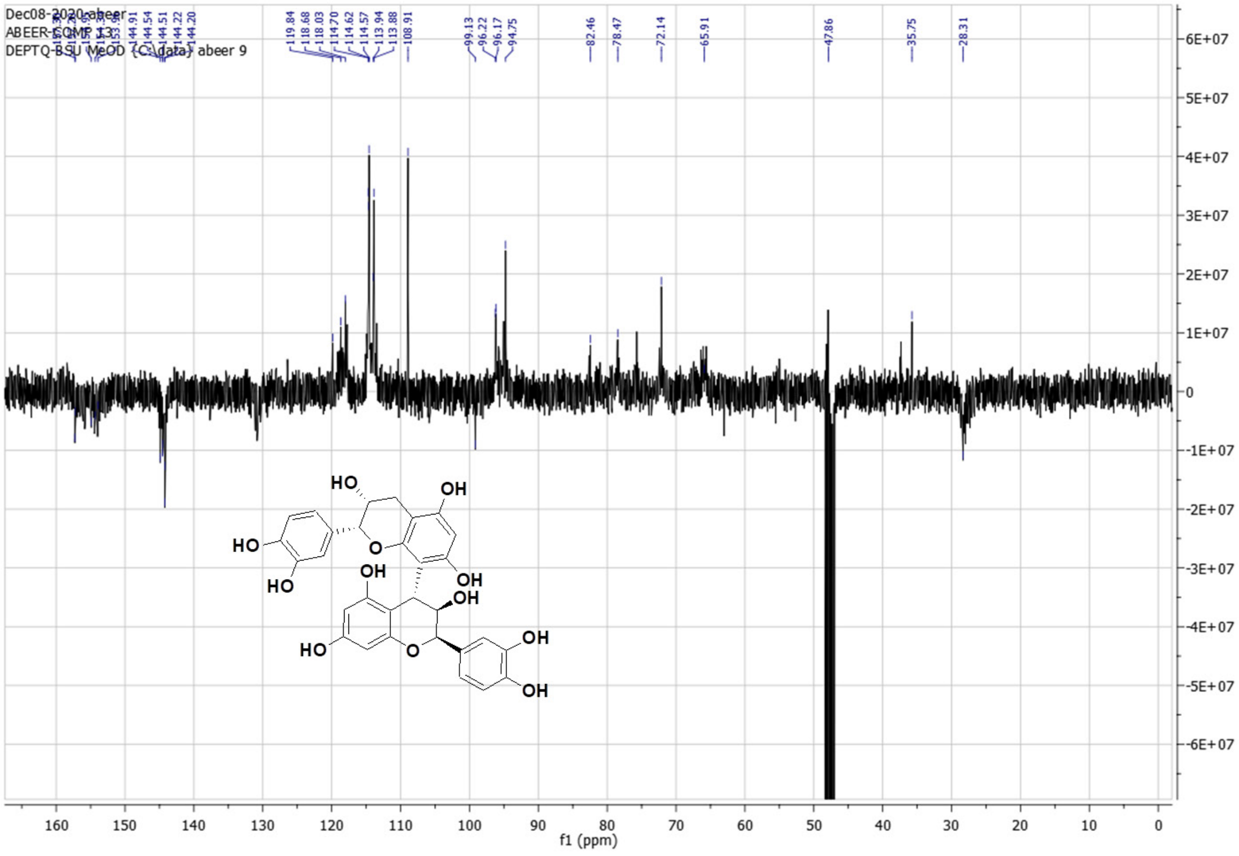

Figure S15. DEPT-Q NMR spectrum of compound **33** measured in CD_3OD-d_4 at 100 MHz

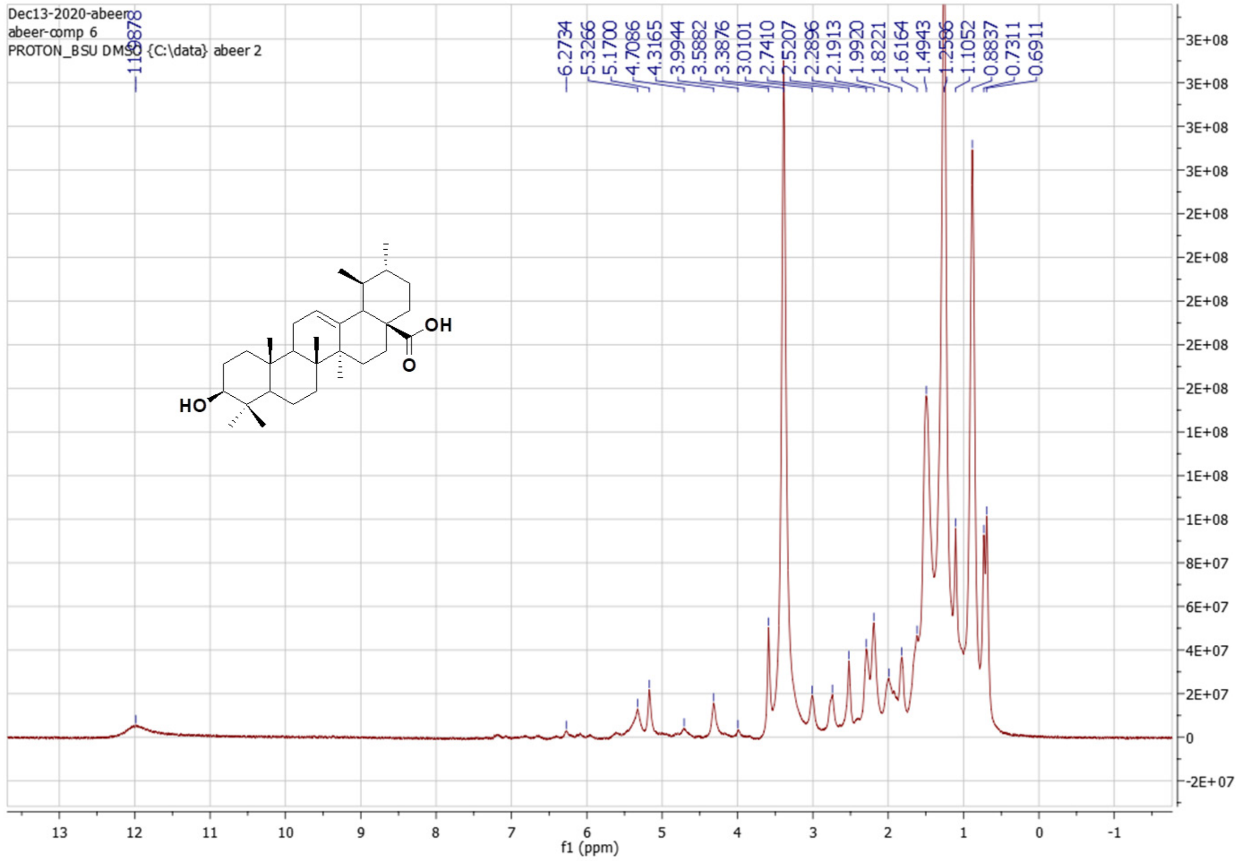

Figure S16. ^{1}H NMR spectrum of compound **34** measured in DMSO-d_6 400 MHz

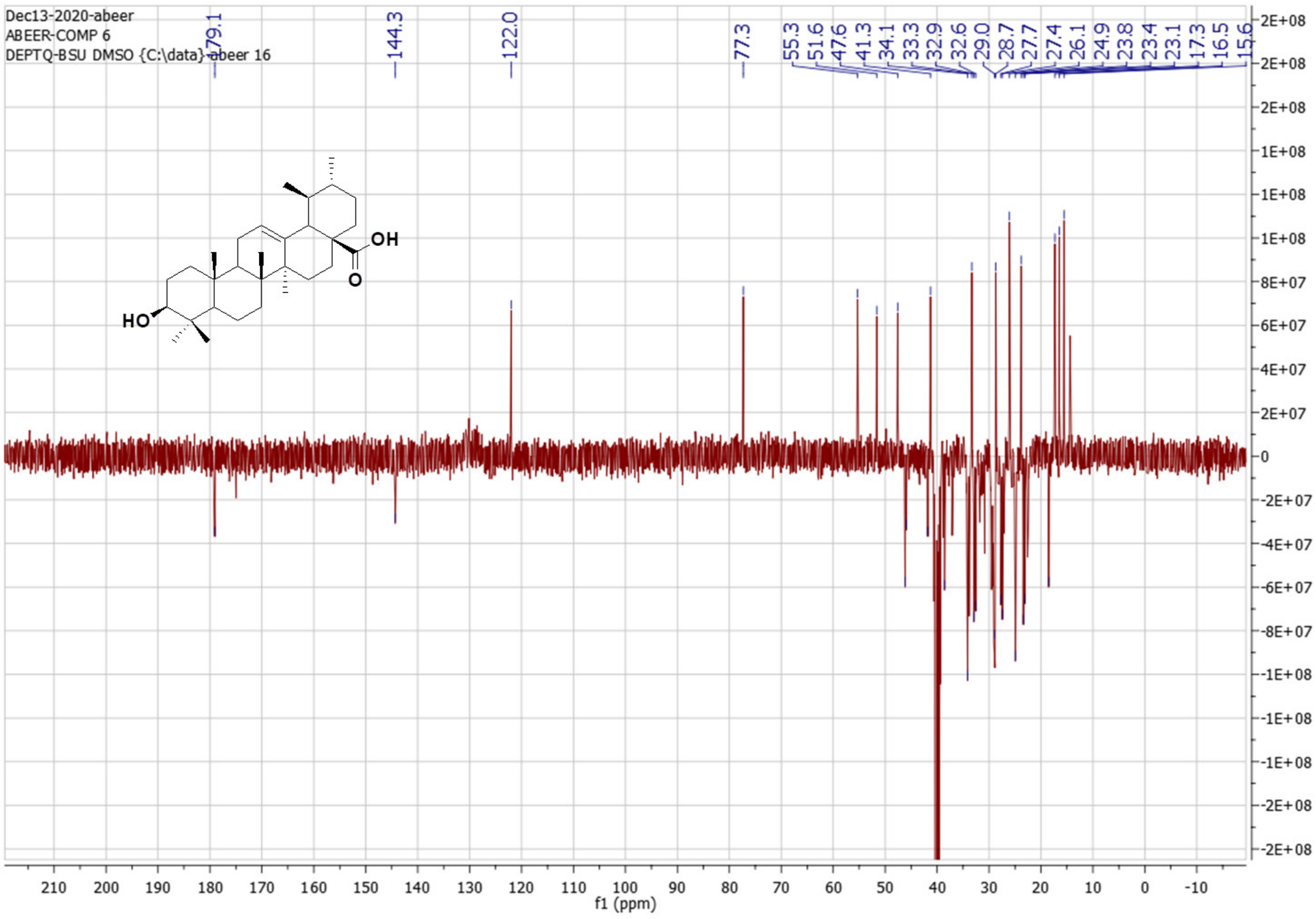

Figure S17. DEPT-Q NMR spectrum of compound **34** measured in DMSO-d_6 at 100 MHz

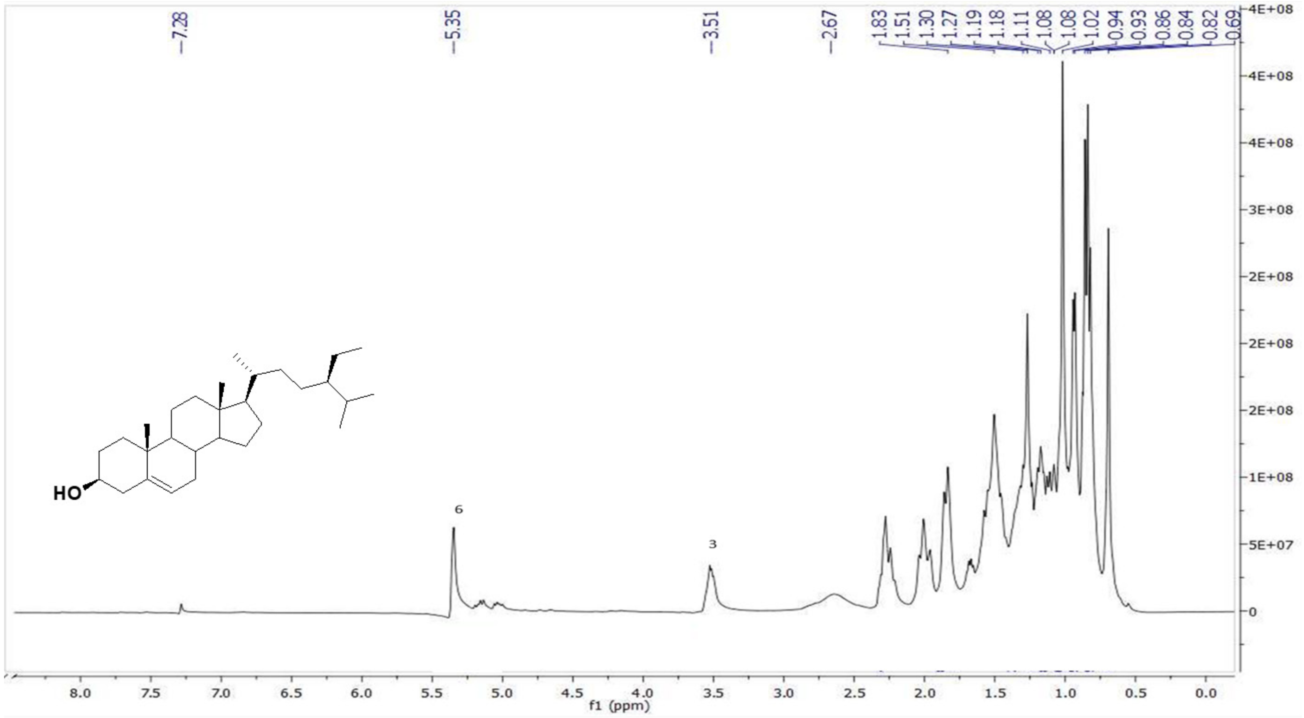

Figure S18. ^{1}H NMR spectrum of compound **35** measured in $CDCL_3$ at 400 MHz

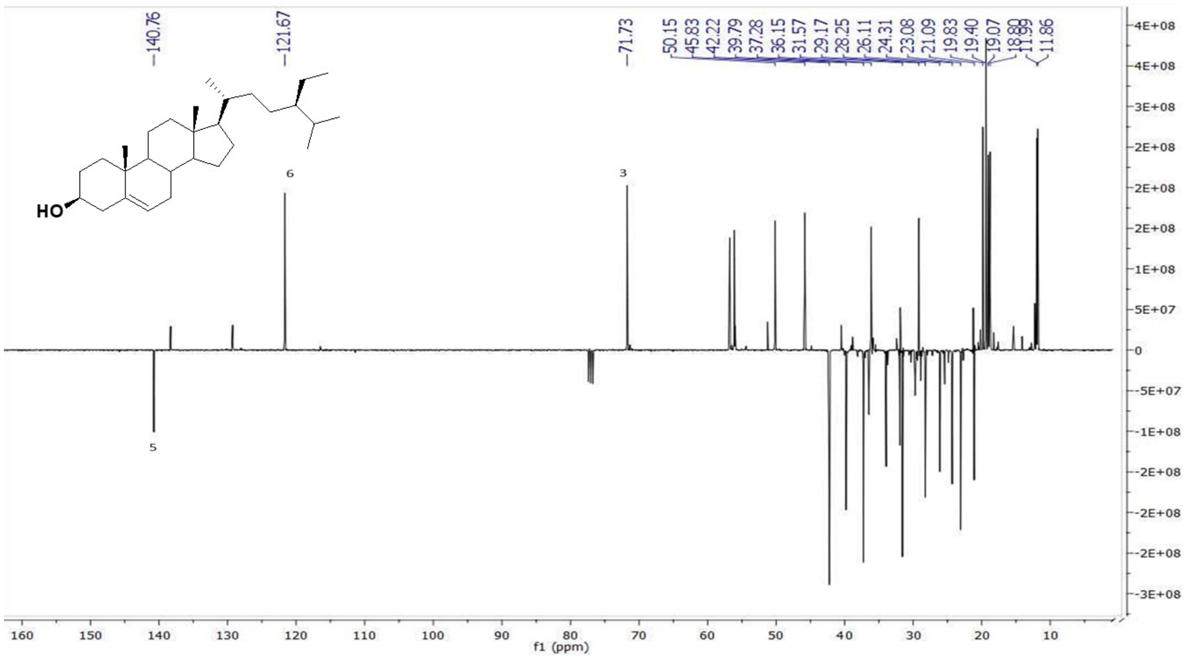

Figure S19. DEPT-Q NMR spectrum of compound **35** measured in $CDCl_3$ at 100 MHz

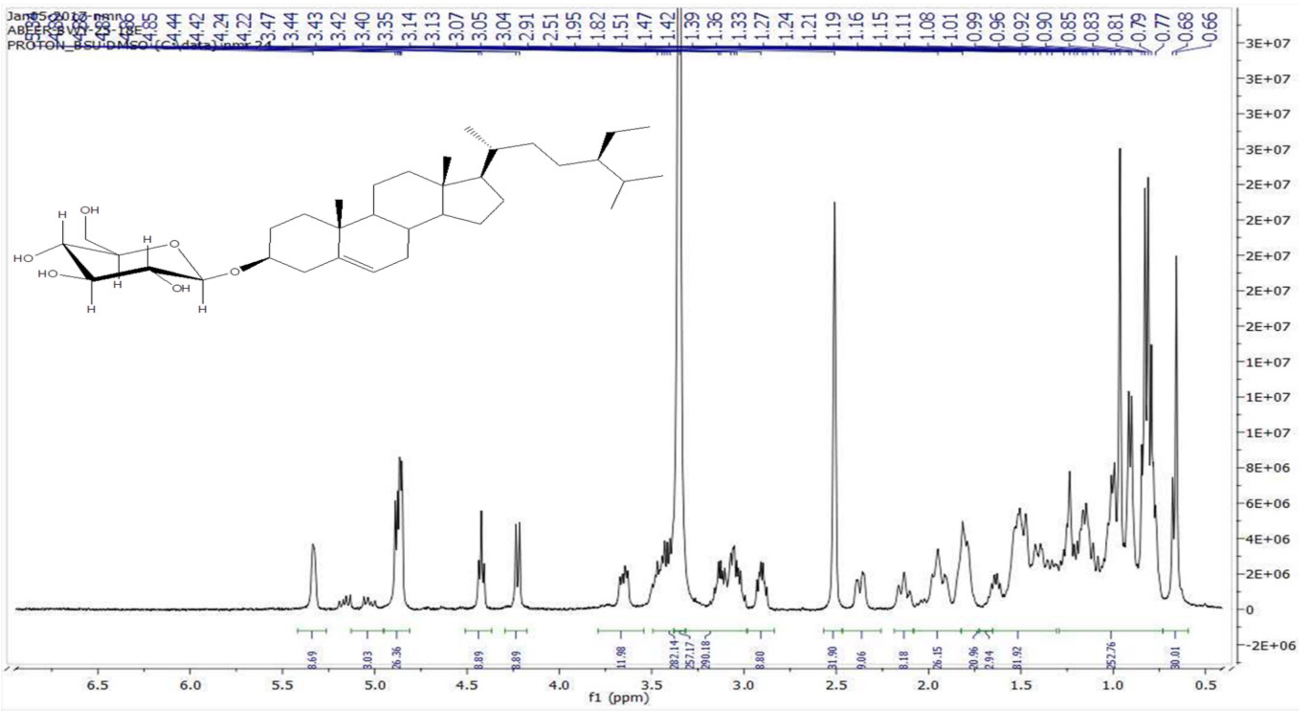

Figure S20. ^{1}H NMR spectrum of compound **36** measured in DMSO-d_6 at 400 MHz

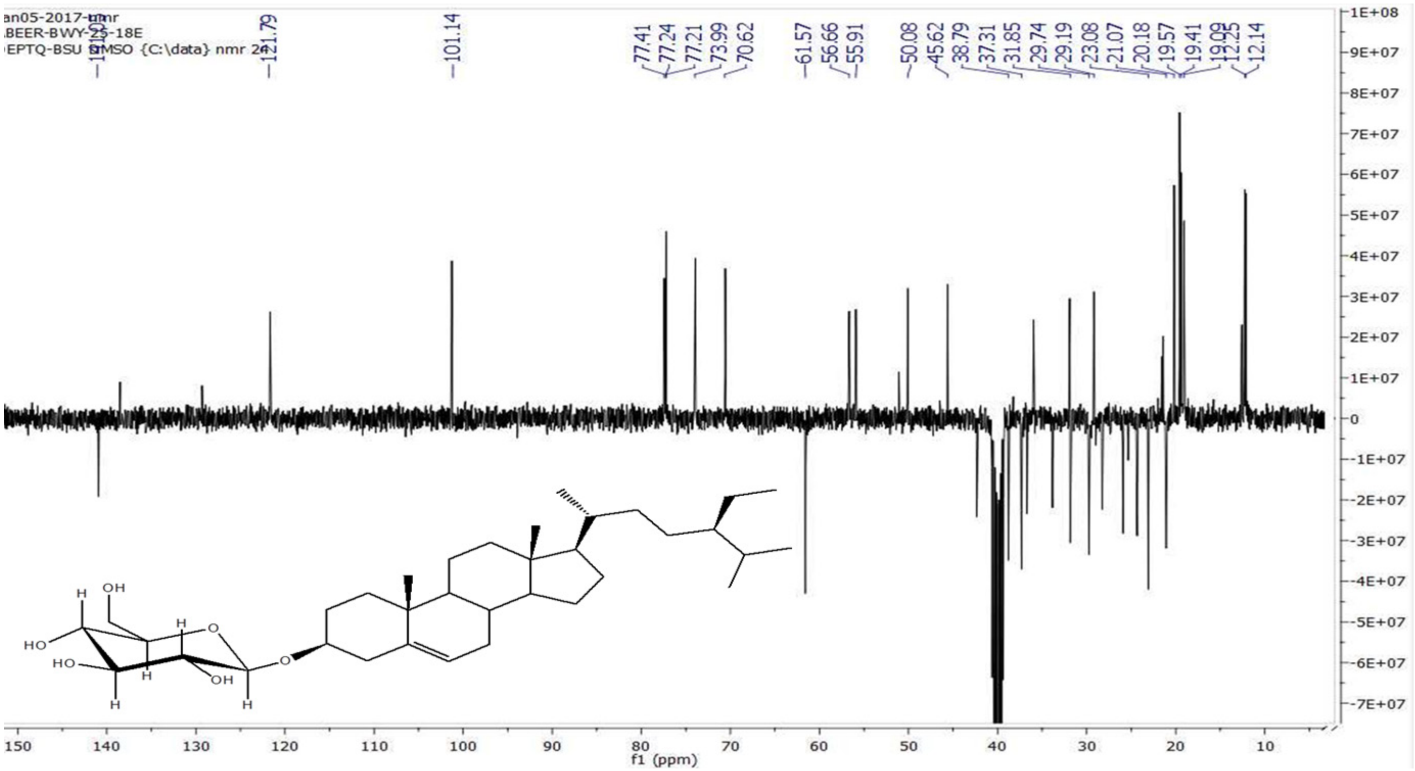

Figure S21. DEPT-Q NMR spectrum of compound **36** measured in DMSO-d_6 at 100 MHz

References

1. Bolliger, H.R.; Brenner, M.; Gänshirt, H.; Mangold, H.K.; Seiler, H.; Stahl, E.; Waldi, D. *Thin-Layer Chromatography: A Laboratory Handbook*; Springer: 1965.
2. Harikrishnan, L.S.; Warrier, J.; Tebben, A.J.; Tonukunuru, G.; Madduri, S.R.; Baligar, V.; Mannoori, R.; Seshadri, B.; Rahaman, H.; Arunachalam, P. Heterobicyclic inhibitors of transforming growth factor beta receptor I (TGFβRI). *Bioorg. Med. Chem.* **2018**, *26*, 1026-1034.
3. Tramontina, V.A.; Machado, M.A.N.; Filho, G.d.R.N.; Kim, S.H.; Vizzioli, M.R.; Toledo, S. Effect of bismuth subgallate (local hemostatic agent) on wound healing in rats. Histological and histometric findings. *Brazilian dental journal* **2002**, *13*, 11-16.
4. Hummon, A.B.; Lim, S.R.; Difilippantonio, M.J.; Ried, T. Isolation and solubilization of proteins after TRIzol® extraction of RNA and DNA from patient material following prolonged storage. *Biotechniques* **2007**, *42*, 467-472.
5. Alsenani, F.; Ashour, A.M.; Alzubaidi, M.A.; Azmy, A.F.; Hetta, M.H.; Abu-Baih, D.H.; Elrehany, M.A.; Zayed, A.; Sayed, A.M.; Abdelmohsen, U.R. Wound Healing Metabolites from Peters' Elephant-Nose Fish Oil: An In Vivo Investigation Supported by In Vitro and In Silico Studies. *Mar. Drugs* **2021**, *19*, 605.
6. Alzarea, S.I.; Elmaidomy, A.H.; Saber, H.; Musa, A.; Al-Sanea, M.M.; Mostafa, E.M.; Hendawy, O.M.; Youssif, K.A.; Alanazi, A.S.; Alharbi, M. Potential anticancer lipoxygenase inhibitors from the red sea-derived brown algae sargassum cinereum: an in-silico-supported In-Vitro Study. *Antibiotics* **2021**, *10*, 416.
7. He, M.M.; Smith, A.S.; Oslob, J.D.; Flanagan, W.M.; Braisted, A.C.; Whitty, A.; Cancilla, M.T.; Wang, J.; Lugovskoy, A.A.; Yoburn, J.C. Small-molecule inhibition of TNF-α. *Science* **2005**, *310*, 1022-1025.
8. Shivananda Nayak, B.; Dan Ramdath, D.; Marshall, J.R.; Isitor, G.; Xue, S.; Shi, J. Wound-healing properties of the oils of Vitis vinifera and Vaccinium macrocarpon. *Phytother. Res.* **2011**, *25*, 1201-1208.
9. Hassan H, Abdel-Aziz A. Evaluation of free radical-scavenging and anti-oxidant properties of black berry against fluoride toxicity in rats. *Food and chemical toxicology.* 2010;48(8-9):1999-2004.
10. Sonboli, A.; Mojarrad, M.; Ebrahimi, S.N.; Enayat, S. Free Radical Scavenging Activity and Total Phenolic Content of Methanolic Extracts from Male Inflorescence of Salix aegyptiaca Grown in Iran. Iran. J. Pharm. Res. IJPR 2010, 9, 293–296.
